# Single and multi-site CT-based radiogenomics analysis of metastatic lung adenocarcinoma and correlations with outcome

**DOI:** 10.1007/s00330-025-12292-8

**Published:** 2026-01-16

**Authors:** Amandine Crombé, Lou Andrea Sitruk, Cécile Masson-Grehaigne, Mathilde Lafon, Jean Palussiere, Benjamin Bonhomme, Sophie Cousin, Nathalie Lassau, Antoine Italiano

**Affiliations:** 1 Department of Diagnostic Oncologic Imaging, Gustave Roussy Institute, Villejuif, France; 2 Laboratoire d’Imagerie Biomedicale Multimodale Paris-Saclay (BioMAPS), Université Paris-Saclay, Inserm, Centre National de la Recherche Scientifique, Commissariat à l’Energie Atomique, Villejuif, France; 3 Department of Radiology, Pellegrin University Hospital, Bordeaux, France; 4 Department of Medical Oncology, Institut Bergonié, Bordeaux, France; 5 Department of Radiology, Institut Bergonié, Bordeaux, France; 6 Department of Pathology, Institut Bergonié, Bordeaux, Bordeaux, France; 7 SARCOTARGET Team, Bordeaux Research Institute in Oncology (BRIC) INSERM U1312 and University of Bordeaux, Bordeaux, France

**Keywords:** Multidetector computed tomography, Adenocarcinoma of lung, High-throughput nucleotide sequencing, Neoplasm metastasis, Radiomics

## Abstract

**Objectives:**

Radiogenomic studies have mostly linked single-site radiomic features (RFs) to genomic alterations in locally-advanced lung cancer, limiting their applicability to patients with metastatic lung adenocarcinoma (MLUAD). Our aim was to evaluate associations between unsupervised CT-based radiomic clustering of single-site and multi-site features and oncogenic alterations (OAs) and response to treatment in MLUAD.

**Materials and methods:**

Patients managed at our center (October 2016–January 2024) with pre-treatment CT scans and next-generation sequencing were retrospectively included. Reproducible RFs were extracted from all solid tumor lesions > 1 cm³ using an automated pipeline. Patient-level integration used the centroid of each patient’s lesions in radiomic space, providing multi-site radiomics data. RFs from the largest and biopsied lesions were also isolated. Patients were clustered by unsupervised hierarchical consensus clustering using centroid-based (Cluster-C), largest lesion (Cluster-M), and biopsied lesion (Cluster-B) features. Uni- and multivariable associations with OAs (any OA, smoker-related [sOA], non-smoker-related [nsOA], or wild-type), overall response rate (ORR), and overall survival (OS) were investigated.

**Results:**

Among 361 patients (median age 63.2 years; 41.3% women; 1721 segmented tumor lesions), 48.2% had sOA and 13% had nsOA. Cluster-M2 + M5 was enriched in KRAS (*p* = 0.048), MET (*p* = 0.046), and PI3KCA (*p* < 0.001) alterations. Cluster-M (especially Cluster-M2 + M5) independently predicted sOA (OR = 2.28, *p* = 0.006), and nsOA (OR = 5.49, *p* = 0.004). Cluster-M was linked to higher ORR (*p* = 0.026) and longer OS (*p* = 0.016).

**Conclusion:**

Baseline CT-based single- and multi-site radiomics capture patterns associated with key OAs in MLUAD, suggesting their potential role as a non-invasive adjunct to guide molecular testing and optimize treatment selection.

**Key Points:**

***Question*** *In MLUAD, can single- and multi-site RFs from all measurable lesions enhance the detection of key OAs and outcome prediction beyond standard clinical–radiological assessment?*

***Findings*** *In 361 MLUAD patients, robust clustering using RFs from multiple tumor lesions per patient identified subgroups associated with key OAs, response to treatment, and survival*.

***Clinical relevance*** *Whatever the initial disease staging, radiomic clustering may serve as a non-invasive AI biomarker that complements molecular testing, helping identify actionable tumor profiles and stratify patients for treatment selection and prognostication in MLUAD*.

**Graphical Abstract:**

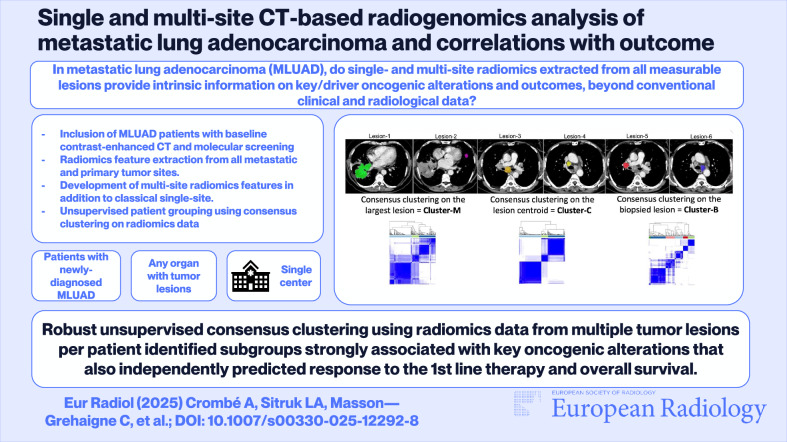

## Introduction

Lung cancer remains the leading cause of cancer-related mortality worldwide, accounting for 1.8 million deaths annually, with a 5-year overall survival (OS) at 27% in 2025 projections [1]. Lung adenocarcinoma (LUAD) is the most common histological subtype, advanced or metastatic at diagnosis, for 70% of patients [2]. The advent of targeted therapies and immune checkpoint inhibitors has dramatically improved outcomes. Specific approaches are thus available in first-line, based on the oncogenic profile, evaluated on tumoral tissue. In this context, a non-invasive imaging biomarker capable of accurately predicting oncogenic alterations (OAs) could reduce the need for repeat biopsies and optimize resource allocation in precision oncology.

Contrast-enhanced CT is the standard imaging modality for metastatic lung adenocarcinoma (MLUAD), and prior studies have linked certain radiological patterns to specific gene alterations [3–5]. Radiomics is a quantitative imaging approach enabling a semi-automated extraction of tumor radiophenotypes via numerical features describing shape and texture [6]. Radiomic features (RFs) can be used in supervised machine learning to predict clinical outcomes or in unsupervised analyses to uncover latent imaging patterns. In lung cancer, single-site radiomics has been used to predict mutational status and OS [7–10]. Recently, Pérez-Johnston et al applied unsupervised clustering to radiomic data from 219 patients with stage I LUAD, revealing significant associations between imaging clusters and *EGFR*, *PI3KCA*, and *STK11* alterations, along with links to histopathologic features and recurrence risk [11].

A major current challenge lies in developing robust methods to integrate radiomic data across multiple tumor sites and metastatic patterns/features. Building on the work of Perez-Johnston et al, the objective of the present study was to extend radiomics-based patient stratification to individuals with stage IIIB–IV MLUAD by applying unsupervised clustering to CT-derived RFs. Both classical single-site radiomics (from the largest and biopsied lesions) and multi-site radiomics were incorporated. The aim was to assess whether these imaging-derived clusters remained associated with key OAs. Because LUAD in non-smokers and smokers displays distinct tumorigenic pathways, with less molecular heterogeneity in the former, these studies were stratified according to patient smoking histories. Moreover, clusters were evaluated with regard to clinical outcomes, including treatment response and OS.

## Materials and methods

### Study design and patients

This retrospective single-center study was IRB-approved (Bergonié Institute, Bordeaux, France). Written informed consent was waived by the IRB due to its retrospective nature.

**Fig. 1:**
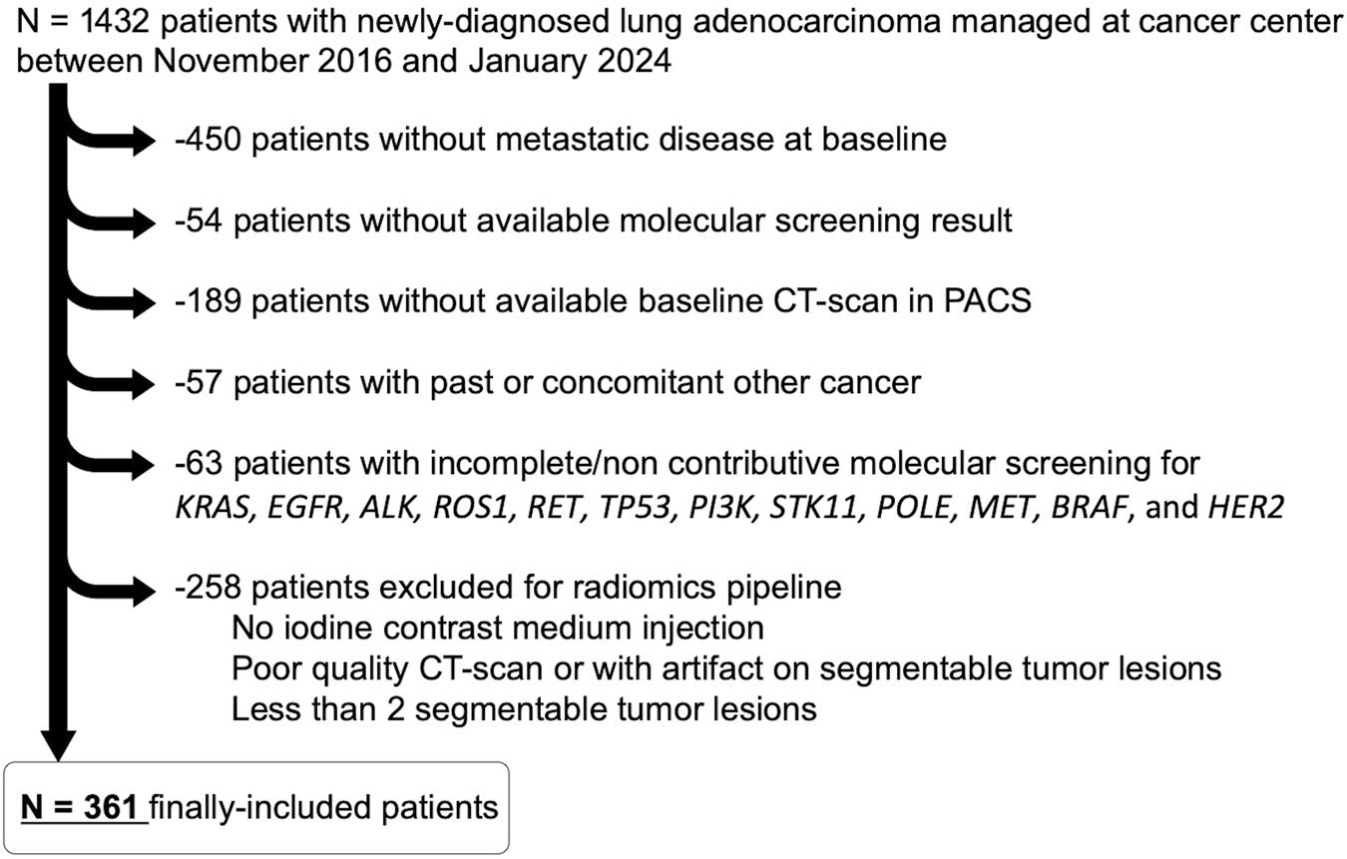
Study flow chart. PACS, picture archiving and communication system

All consecutive adults with newly-diagnosed LUAD between October 2016 and January 2024 addressed to our regional comprehensive cancer center (Bergonié Institute) were included if they fulfilled inclusion criteria, i.e., good quality contrast-enhanced whole-body CT-scan including a portal phase, metastatic disease at baseline, at least two segmentable lesions ≥ 1 cm^3^, routine molecular screening, and no other concomitant cancer. Patients were identified through the pathology database (Fig. 1). There is a partial overlap with a prior published cohort with distinct study objectives and statistical approach [12].

Age, sex, World Health Organization performance status (WHO-PS), tobacco addiction (non-smoker, smoker, and past smoker), staging, and first-line treatment were collected.

Clinical outcomes were overall response rate (ORR) to first-line therapy according to response evaluation criteria in solid tumors v1.1 [13], and OS, defined as time from the date of diagnosis to the date of disease-related death, or last follow-up, censoring patients lost to follow-up at the end of data collection (February 2024), or with death unrelated to cancer.

### Pre-treatment biopsy pathology and molecular data

Programmed-death ligand 1 (PD-L1) expression status was categorized as 0%, 1–49%, and 50–100% positivity.

Molecular profiling was routinely performed on pre-treatment tumor samples using a next-generation sequencing panel recommended by French national guidelines comprising alterations in *KRAS, EGFR, ALK, ROS1, RET, TP53, PI3K, STK11, POLE, MET, BRAF*, and *HER2* [14]. Patients were defined as ‘true wild-type’ if no alteration was detected in any of the aforementioned genes. OAs were then categorized as follows:

(i) ‘Any OA’ included alterations in *KRAS, EGFR, ALK, ROS1, RET, PI3K, STK11, POLE, MET, BRAF*, or *HER2*.

(ii) ‘Smoker-related OA’ (sOA) included alterations in *BRAF, KRAS, STK11*, or *MET*.

(iii) ‘Non-smoker-related OA’ (nsOA) included *EGFR, ALK*, or *ROS1* alterations, in the absence of co-occurring *BRAF, KRAS, STK11*, or *MET* mutations.

Alterations in *TP53* were not included in OA definitions due to their high prevalence across multiple cancer types, limited specificity as actionable drivers, and unclear predictive value for targeted therapies. *TP53* mutations are widely regarded as indicators of genomic instability rather than as actionable oncogenic events [15].

### CT acquisition and radiological analysis

Pre-treatment contrast-enhanced whole-body CT-scans were acquired using multiple scanning systems (Canon, Siemens Healthineers, General Electric Healthcare, Philips Healthcare) from several private and public radiological centers from the Nouvelle-Aquitaine region in France. In-plane resolution ranged from 0.5 × 0.5 mm² to 1 × 1 mm², and slice thickness between 0.6 and 2.5 mm. Brain lesions were acquired at delayed phase (5 min post-contrast) while thorax-abdomen-pelvic lesions were acquired at the portal phase (70 s post-contrast).

CT images were reviewed by two radiologists blinded to clinical-pathological data, A.C., a senior with 8 years of experience, and L.A.S., a fellow with 6 months of training in oncologic imaging.

For each patient, the metastatic pattern was assessed by recording the presence/absence of metastases in the lung (including miliary pattern), pleura, pericardium, brain, leptomeninges, liver, spleen, kidney, adrenals, bone, muscle and skin, as well as epiduritis (isolated or associated with vertebral metastases), carcinomatous lymphangitis, peritoneal or retroperitoneal carcinomatosis, N3 or N4 lymphadenopathy. Radiologists had access to ^18^F-fluorodeoxyglucose positron emission tomography, CT, and follow-up CT scans when needed.

### Radiomics pipeline

The radiomics workflow only involved images in the abdominal kernel and comprised CT conversion from DICOM to nifti format, voxel size standardization, gray level discretization and normalization, 3D-manual segmentation of each measurable lesion ≥ 1 cm^3^ (including two segmentations for 100 randomly sampled tumors) [16, 17]. RFs were then selected if their inter-segmentation intra-class correlation coefficient was > 0.85 with a non-near-zero variance. RF transformation included center-scaling then Yeo-Johnson transformation to normalize their distribution [18]. Details about the segmentation technique, inter-observer reproducibility analysis, image post-processing (including voxel size standardization and gray level discretization), RF extraction, filtering, and normalization are provided in Supplemental Method SM1.

### Radiomics clusters

**Fig. 2:**
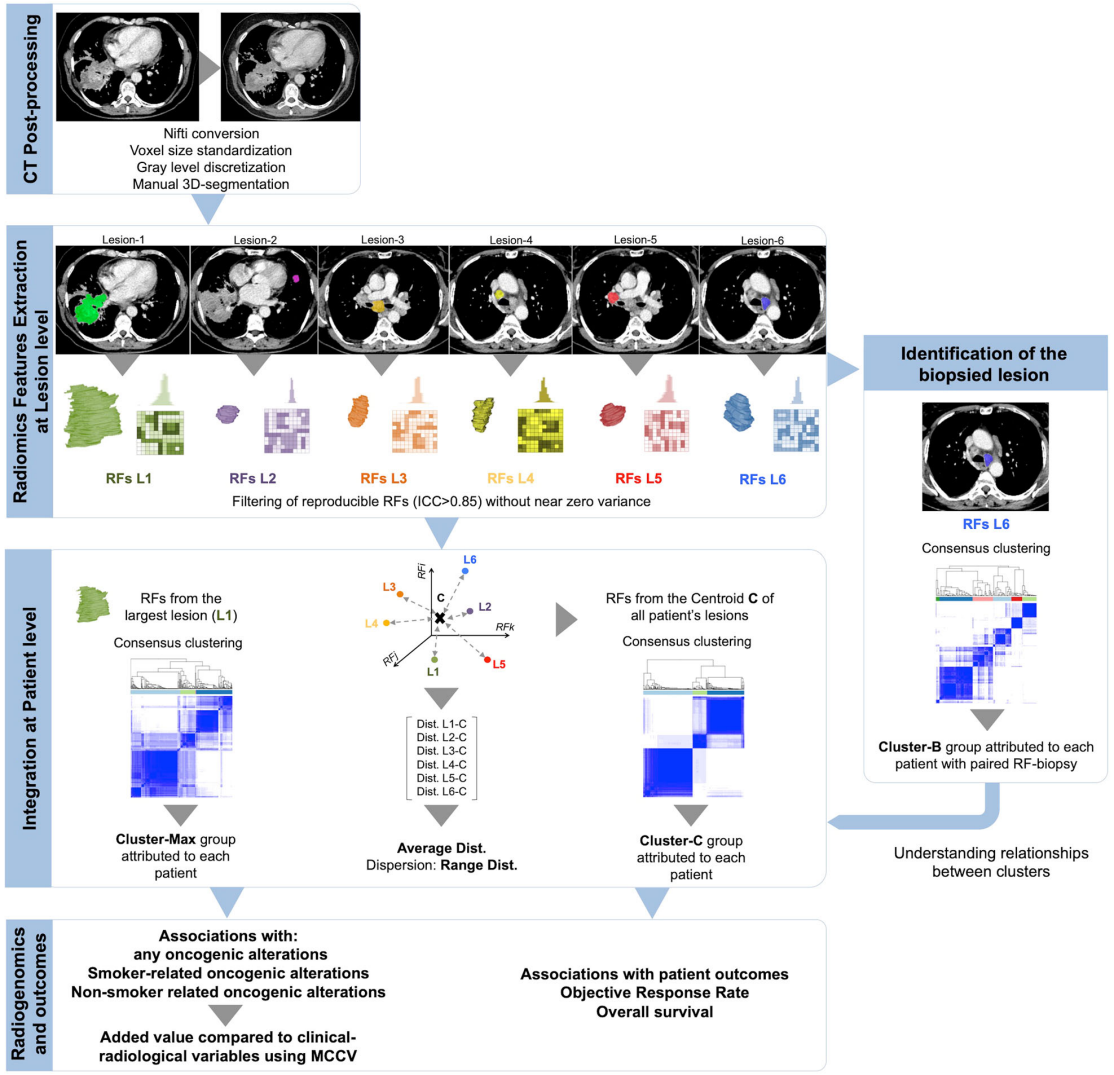
Study workflow. The four panels of the workflow illustrate the following main steps: (i) CT post-processing to harmonize RFs across patients; (ii) feature extraction from each solid, segmentable tumor lesion for each patient (e.g., RFs for lesion 1 [RFs L1], lesion 2 [RFs L2], lesion 6 [RFs L6]…), followed by filtering for reproducibility and informativeness using intra-class correlation coefficient (ICC) > 0.85) and exclusion of features with near-zero variance; and (iii) patient-level integration of lesion-level data, either by selecting RFs from the largest lesion or by averaging RFs across all lesions to define each patient radiomic centroid (C). From this centroid, Euclidean distances to each lesion were calculated, allowing computation of the average and range of lesion-to-centroid distances. When available, RFs from the biopsied lesion used for molecular screening were also included. Hierarchical consensus clustering was then applied to derive three patient-level clusters: Cluster-M, Cluster-C, and Cluster-B, respectively; (iv) interpretation of clusters, focusing on their relationships with OAs and clinical outcomes. MCCV, Monte Carlo cross-validation; RF, radiomics feature

To integrate radiomic information from multiple lesions into a single patient-level representation, methodological approaches were developed (Supplemental Material SM2), as summarized in Fig. 2, allowing to establishment of clusters based on hierarchical consensus clustering [19]. Centroid-based clustering (Cluster-C) took into account a single centroid for each patient, who was then assigned to the optimal cluster identified through this procedure. Largest lesion-based clustering (Cluster-M), considered the most important lesion for each patient, was again associated with numbers representing sub-clusters derived from this single-lesion approach. Biopsied-lesion clustering (Cluster-B) used pre-biopsy RFs, whenever available. To complement these unsupervised approaches, we computed the Euclidean distance between each lesion and its corresponding patient-level centroid in the RF space. The mean and range of these distances were then calculated for each patient, serving as quantitative proxies for the spatial dispersion and heterogeneity of radiomic profiles among lesions.

### Statistical analyses

Statistical analyses were performed with R (v4.1.0). All tests were two-tailed. *p-*values < 0.05 defined statistical significance.

Characteristics were compared across different groups using Chi-Square tests for categorical variables and Kruskal-Wallis tests for non-normal numeric variables. Continuous variables were compared using Student *t*- or Mann-Whitney tests after checking normality by the Shapiro–Wilk test. Univariable and multivariable analyses were performed, using variables with *p* < 0.05 in the former, for stepwise selection to identify independent predictors of OAs. Odds ratios (ORs) and 95% confidence intervals (CIs) were reported for both univariable and multivariable analyses. OS was analyzed using Kaplan–Meier estimates and compared between groups using the log-rank test. Univariable Cox proportional hazards models were used to estimate hazard ratios (HRs) and 95% CIs. Analyses were performed in the overall cohort and stratified by OA type. Multivariable Cox regression models were constructed by including the radiomics-derived features significant in univariable analysis together with key clinical covariates (age, sex, WHO-PS, and TNM stage).

Between-clusters similarities were investigated with adjusted Rand Index (ARI) for the entire population, as well as for patients with wild-type, nsOA, and sOA (Supplemental Material SM3) [20].

Three binary classification tasks were modeled to assess whether RFs enhanced the prediction of OA subtypes. Model performances were assessed on the out-of-bag independent test sets from a 1000-fold repeated Monte Carlo cross-validation (i.e., in order to avoid overfitted results), according to the area under the ROC curve (AUROC). Average AUROC and 95% CI across the 1000 iterations were used to estimate predictive performance and identify the most discriminative combination of input features. Patients with missing data were excluded from multivariable analysis.

A complete list of packages and versions is provided in Supplementary Methods SM4. The R script can be found at https://github.com/ACrombe/HIR-Mutations. Supplementary Methods 5 shows the corresponding checklist for artificial intelligence in medical imaging (CLAIM).

## Results

### Patient and tumor lesion characteristics

Overall, 361 patients with MLUAD, with a median age of 63.2 years (IQR 56–69.6), 149 of them (41.3%) being women, were included (Table 1). Most were current (167/357, 46.8%) or former smokers (149/357, 41.7%). The most frequent genomic alterations involved *KRAS* (145/361, 40.2%), *TP53* (88/361, 24.4%), and *EGFR* (39/361, 10.8%).

**Table 1:** Patient characteristics

Characteristics	Patients
Age (years)	63.2 [56–69.6] (22–87.9)
Sex (women)	149/361 (41.3)
Tobacco addiction	
Never smoker	41/357 (11.5)
Active smoker	167/357 (46.8)
Former smoker	149/357 (41.7)
WHO PS	
0	97/361 (26.9)
1	190/361 (52.6)
≥ 2	74/361 (20.5)
Staging	
III–IVa	82/361 (22.7)
IVb	279/361 (77.3)
No. of distinct metastatic organs	
1	73/361 (20.2)
2	96/361 (26.6)
≥ 3	192/361 (53.2)
Metastasis locations on initial imaging	
Lung	154/361 (42.7)
Leptomeningeal carcinomatosis	3/361 (0.8)
Epiduritis	30/361 (8.3)
Brain	111/361 (30.7)
Pericardial	16/361 (4.4)
Miliary	10/361 (2.8)
Carcinomatous lymphangitis	47/361 (13)
Pleura	70/361 (19.4)
Mucle	40/361 (11.1)
Spleen	12/361 (3.3)
Kidney	12/361 (3.3)
Pancreas	10/361 (2.8)
N3 or N4 lymphadenopathy	228/361 (63.2)
Liver	77/361 (21.3)
Adrenal	120/361 (33.2)
Subcutaneous	26/361 (7.2)
Peritoneal carcinomatosis	33/361 (9.1)
Bone	172/361 (47.6)
PD-L1 TPS score	
0%	135/343 (39.4)
1–49%	101/343 (29.4)
50–100%	107/343 (31.2)
Gene alterations at initial screening	
* ROS1*	3/361 (0.8)
* HER2*	9/361 (2.5)
* ALK*	7/361 (1.9)
* KRAS*	145/361 (40.2)
* TP53*	88/361 (24.4)
* EGFR*	39/361 (10.8)
* BRAF*	18/361 (5)
* MET*	6/361 (1.7)
* POLE*	0/361 (0)
* STK11*	28/361 (7.8)
* PI3KCA*	10/361 (2.8)

Data are numbers of patients with percentage in parentheses, except for age, given as median, interquartile range, and range. *TPS* tumor proportion score, *WHO-PS* World Health Organization performance status. Smoker status and PD-L1 TPS were unavailable for 4 and 18 patients, respectively

Based on genomic profiles, 92/361 (25.5%) patients were wild-type, 174/361 (48.2%) had sOA, and 47/361 (13%) had nsOA.

Table 2 details the characteristics of these OA subgroups, highlighting the influence of sex, tobacco consumption, WHO-PS, and type of metastasis (brain, pleura, peritoneal, and bone), as well as sub-clusters on the types of OAs.

**Table 2:** Associations between clinical, radiological, and radiomics data with patient status according to molecular screening

Characteristics	WT patients (*N* = 92)	Patients with at least 1 OA (*N* = 233)	Patients with sOA (*N* = 174)	Patients with nsOA (*N* = 47)	WT vs at least 1 OA *p*-value	WT vs sOA *p*-value	WT vs nsOA *p*-value	sOA vs nsOA *p*-value
Age (years)	64.2 [58–69.6] (22–87.9)	63.1 [54.4–69.8] (26–83.7)	63.1 [55.6–69.8] (25–83.7)	61.9 [54.1–69.5] (25–83.2)	0.155	0.224	0.188	0.670
Sex (women)	29/92 (31.5)	108/233 (46.4)	68/174 (39.1)	31/47 (66)	0.021*	0.278	**<** 0.001***	0.002**
Tabacco addiction					0.218	0.260	**<** 0.001***	**<** 0.001***
Never smoker	7/90 (7.8)	34/231 (14.7)	6/172 (3.5)	25/47 (53.2)				
Active smoker	42/90 (46.7)	106/231 (45.9)	91/172 (52.9)	9/47 (19.1)				
Past smoker	41/90 (45.6)	91/231 (39.4)	75/172 (43.6)	13/47 (27.7)				
WHO PS					0.255	0.752	0.029*	0.062
0	22/92 (23.9)	64/233 (27.5)	44/174 (25.3)	17/47 (36.2)				
1	45/92 (48.9)	125/233 (53.6)	90/174 (51.7)	26/47 (55.3)				
≥ 2	25/92 (27.2)	44/233 (18.9)	40/174 (23)	4/47 (8.5)				
Staging					0.905	> 0.999	0.379	0.316
III–IVa	21/92 (22.8)	50/233 (21.5)	40/174 (23)	7/47 (14.9)				
IVb	71/92 (77.2)	183/233 (78.5)	134/174 (77)	40/47 (85.1)				
No. of distinct metastatic organs					0.958	0.908	0.604	0.477
1	18/92 (19.6)	43/233 (18.5)	35/174 (20.1)	6/47 (12.8)				
2	25/92 (27.2)	62/233 (26.6)	43/174 (24.7)	14/47 (29.8)				
≥ 3	49/92 (53.3)	128/233 (54.9)	96/174 (55.2)	27/47 (57.4)				
Metastasis locations on initial imaging								
Lung	37/92 (40.2)	104/233 (44.6)	79/174 (45.4)	19/47 (40.4)	0.549	0.496	> 0.999	0.6571
Leptomeningeal carcinomatosis	0/92 (0)	2/233 (0.9)	1/174 (0.6)	1/47 (2.1)	-	-	-	-
Epiduritis	7/92 (7.6)	20/233 (8.6)	14/174 (8)	5/47 (10.6)	0.949	> 0.999	0.778	0.788
Brain	26/92 (28.3)	74/233 (31.8)	47/174 (27)	23/47 (48.9)	0.630	0.942	0.026*	0.007*
Pericardial	5/92 (5.4)	11/233 (4.7)	10/174 (5.7)	1/47 (2.1)	-	-	-	-
Miliary	2/92 (2.2)	8/233 (3.4)	3/174 (1.7)	3/47 (6.4)	-	-	-	-
Carcinomatous	10/92 (10.9)	29/233 (12.4)	17/174 (9.8)	10/47 (21.3)	0.838	0.945	0.162	0.059
Pleural	11/92 (12)	53/233 (22.7)	40/174 (23)	11/47 (23.4)	0.040*	0.044*	0.133	> 0.999
Mucle	7/92 (7.6)	31/233 (13.3)	29/174 (16.7)	2/47 (4.3)	0.212	0.062	0.692	0.053
Spleen	1/92 (1.1)	10/233 (4.3)	9/174 (5.2)	1/47 (2.1)	0.272	0.184	> 0.999	0.620–
Kidney	1/92 (1.1)	9/233 (3.9)	8/174 (4.6)	1/47 (2.1)	0.343	0.250	> 0.999	0.731
Pancreas	3/92 (3.3)	7/233 (3)	6/174 (3.4)	1/47 (2.1)	> 0.999	> 0.999	> 0.999	> 0.999
N3 or N4 lymphadenopathy	63/92 (68.5)	145/233 (62.2)	111/174 (63.8)	26/47 (55.3)	0.353	0.530	0.179	0.372
Liver	17/92 (18.5)	57/233 (24.5)	42/174 (24.1)	11/47 (23.4)	0.311	0.367	0.644	> 0.999
Adrenal	31/92 (33.7)	75/233 (32.2)	61/174 (35.1)	10/47 (21.3)	0.897	0.931	0.186	0.105
Subcutaneous	8/92 (8.7)	15/233 (6.4)	15/174 (8.6)	0/47 (0)	0.635	> 0.999	0.080	0.079
Peritoneal carcinomatosis	9/92 (9.8)	21/233 (9)	21/174 (12.1)	0/47 (0)	0.998	0.721	0.064	0.026*
Bone	42/92 (45.7)	116/233 (49.8)	78/174 (44.8)	32/47 (68.1)	0.583	> 0.999	0.020*	0.008*
PD-L1 TPS					0.072	0.076	0.054	0.203
0%	41/87 (47.1)	81/221 (36.7)	57/166 (34.3)	18/44 (40.9)				
1–49%	19/87 (21.8)	77/221 (34.8)	56/166 (33.7)	18/44 (40.9)				
50–100%	27/87 (31)	63/221 (28.5)	53/166 (31.9)	8/44 (18.2)				
Average of Euclidean lesion-to-centroid distances	3.67 [2.91–4.51] (1.35–8.59)	3.65 [3.02–4.55] (0.75–8.50)	3.67 [3.02–4.54] (0.75–8.50)	3.46 [3–4.53] (1.02–7.11)	0.781	0.757	0.781	0.566
Range Euclidean lesion-to-centroid distances	2.84 [1.72–4.16] (0–13.72)	2.65 [1.34–4.20] (0–14.40)	2.76 [1.27–4.51] (0–14.40)	2.16 [1.41–2.85] (0–8.45)	0.289	0.539	0.018*	0.057
Cluster-C					**<** 0.001***	**<** 0.001***	0.005*	0.734
Cluster-C1	28/92 (30.4)	120/233 (51.5)	90/174 (51.7)	**25/47** **(53.2)**				
Cluster-C2	**52/92 (56.5)**	75/233 (32.2)	57/174 (32.8)	13/47 (27.7)				
Cluster-C3	12/92 (13)	38/233 (16.3)	27/174 (15.5)	**9/47** **(19.1)**				
Cluster-M					**<** 0.001***	**<** 0.001***	0.002**	0.026*
ClusterM-1	6/92 (6.5)	39/233 (16.7)	**34/174** **(19.5)**	3/47 (6.4)				
Cluster-M2 + 5	22/92 (23.9)	93/233 (69.2)	63/174 (36.2)	**25/47** **(53.2)**				
Cluster-M3 + 4	**64/92** **(69.6)**	101/233 (75.9)	77/174 (44.3)	19/47 (40.4)				

Data are the number of patients with percentage in parentheses, except for numeric variables given as median, 1st and 3rd quartile range, and minimum-maximum range. *p < 0.05, **p < 0.005, ***p < 0.001. Significant results are in bold

*OA* oncogenic alteration, *RFs* radiomics features, *nsOA* non-smoker oncogenic alteration, *sOA* smoker oncogenic alteration, *TPS* tumor positive score, *WHO-PS* World Health Organization performance status, *WT* wild-type

As a whole, 1721 tumor lesions were segmented with a median of 4 per patient (interquartile range [IQR] 3–6, mean: 4.8 ± 2.6) (Supplemental Table ST1). In 180/361 (49.9%) patients, the lesion biopsied was clearly identified and had available RFs. It was the primary lung tumor in two-thirds of the patients (110/180, 61.1%), forming the basis of Cluster-B.

### Clustering results

The number of optimal clusters varied, according to the classes defined, respectively three for Cluster-C (namely C1, C2, and C3), five for Cluster-M, and six for Cluster-B. The 5 sub-clusters of Cluster-M were grouped in order to obtain the balanced categories of M1, M2 + M5, and M3 + M4, because of low counts in clusters M4 (2/361, 0.6%) and M5 (6/361, 1.7%).

**Fig. 3:**
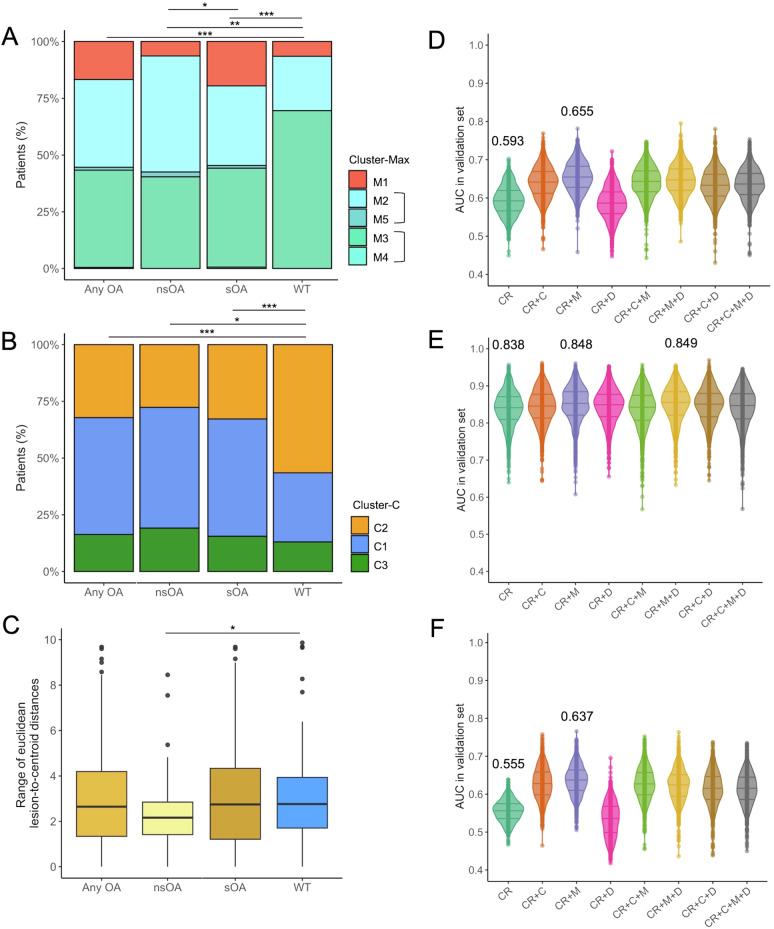
Associations between radiomics features and OA. Distribution of patients across Cluster-M (**A**) and Cluster-C (**B**) stratified by molecular subgroups: any OA, non-smoker OA (nsOA), smoker OA (sOA), and true wild-type (WT). **C** Boxplots showing the range of lesion-to-centroid Euclidean distances across the four molecular subgroups. Distributions of area under the ROC curve (AUC) values from 1000 independent test sets for models predicting: **D** WT vs any OA, **E** WT vs nsOA, **F** WT vs sOA. Models are based on different input features indicated on the *x*-axis: CR, clinical–radiological variables (as selected in the stepwise multivariable analyses presented in Table 3), C Cluster-C, M Cluster-M, D range of Euclidean distances. Horizontal lines in boxplots represent the 1st quartile, median, and 3rd quartile. ^*^p < 0.05, ^**^p < 0.005, ^***^p < 0.001. OA, oncogenic alteration

Figure 3 shows high concordances between radiomic clustering and OAs. Clustering consistency was higher in nsOA patients, indicating more homogeneous radiomic profiles in this subgroup (ARIs were 0.490 for Cluster-B vs Cluster-C; 0.407 for Cluster-B vs Cluster-M; 0.535 for Cluster-C vs Cluster-M) (Supplemental Table 2). There was a lower range (i.e., dispersion) of Euclidean distances in the nsOA group vs wild-type, also consistent with a higher radiophenotypic resemblance between lesions in patients with nsOA (Fig. 3C).

Supplemental Tables 3–5 indicate how sub-clusters in cluster-C, -M, and -B were differentially associated with clinical, pathological, imaging or molecular features, each with individual specificities. One subcluster in each category was more frequent in women; staging impacted cluster-C and cluster-M, while cluster-B5 was associated with more leptomeningeal metastases. From an oncogenic point of view, subclusters in the three categories differentiate by the presence or not of *TP53* and *MET* anomalies.

### Associations with OA groups

Table 3 reports on univariable and multivariable analyses in search of predictors of patient status on the basis of patient characteristics and clustering, using wild-type as reference. This was performed in the three groups of at least one OA, sOA, and nsOA. Additionally, sOA was used as a reference to examine the nsOA group.

**Table 3:** Univariable and multivariable analyses to identify independent predictors of patient status according to initial molecular screening

Characteristics	Univariable OR (95% CI)	*p*-value	Multivariable OR (95% CI)	*p*-value
Any OA vs wild-type (ref.)				
Sex = women (ref.: men)	1.88 (1.13–3.12)	0.015^*^	1.77 (1.05–3.03)	0.035^*^
Pleural metastasis = yes (ref.: no)	2.17 (1.08–4.37)	0.030^*^	1.93 (0.97–4.15)	0.073
Cluster-C				
Cluster-C2 (ref.)	-	-	Removed step 1	
Cluster-C1	2.97 (1.73–5.11)	**<** 0.001^***^		
Cluster-C3	2.2 (1.05–4.6)	0.037^*^		
Cluster-M				
Cluster-M3 + M4 (ref.)	-	-		
Cluster-M1	4.12 (1.65–10.28)	0.002^**^	4.02 (1.70–11.14)	0.003^**^
Cluster-M2 + M5	2.68 (1.53–4.69)	**<** 0.001^***^	2.39 (1.37–4.30)	0.003^**^
sOA vs wild-type (ref.)				
Pleural metastasis = yes (ref.: no)	2.2 (1.07–4.53)	0.032^*^	1.82 (0.88–3.98)	0.116
Cluster-C				
Cluster-C2 (ref.)			Removed step 1	
Cluster-C1	2.93 (1.66–5.17)	**<** 0.001^***^		
Cluster-C3	2.05 (0.94–4.46)	0.070		
Cluster-M				
Cluster-M3 + M4 (ref.)	-	-	-	-
Cluster-M1	4.71 (1.86–11.93)	0.001^**^	4.30 (1.79–12.04)	0.002^**^
Cluster-M2 + M5	2.38 (1.32–4.28)	0.004^**^	2.28 (1.28–4.19)	0.006^*^
nsOA vs wild-type (ref.)				
Sex = women (ref. : men)	4.21 (1.99–8.88)	**<** 0.001^***^	3.56 (1.24–11.03)	0.021^*^
Tobacco addiction				
Active or past smoker (ref.)	-	-		
Never smoker	13.47 (5.15–35.22)	**<** 0.001^***^	22.00 (6.20–97.00)	**<** 0.001^***^
WHO PS				
0 (ref.)	-	-		
1	0.75 (0.34–1.66)	0.474	0.37 (0.10–1.29)	0.126
≥ 2	0.21 (0.06–0.71)	0.012^*^	0.19 (0.03–0.98)	0.057
Brain metastasis = yes (ref.: no)	2.43 (1.17–5.05)	0.017^*^	12.74 (3.45–60.86)	**<** 0.001^***^
Bone metastasis = yes (ref.: no)	2.54 (1.21–5.31)	0.013^*^	6.14 (1.99–22.17)	0.003^**^
Range of Euclidean lesion-to-centroid distances	0.81 (0.66–0.96)	0.023^*^	0.74 (0.51–0.99)	0.065
Cluster-C				
Cluster-C2 (ref.)	-	-	Removed step 1	
Cluster-C1	3.57 (1.58–8.05)	0.002^**^		
Cluster-C3	3 (1.04–8.63)	0.042^*^		
Cluster-M				
Cluster-M3 + M4 (ref.)	-	-	-	-
Cluster-M1	1.68 (0.38–7.38)	0.489	0.99 (0.08–9.29)	0.993
Cluster-M2 + M5	3.83 (1.78–8.25)	**<** 0.001^***^	5.49 (1.78–19.02)	0.004^**^
nsOA vs sOA (ref.)				
Sex = women (ref. : men)	3.02 (1.54–5.94)	0.001^**^	2.77 (1.14–7.03)	0.026^*^
Tobacco addition				
Active or past smoker (ref.)	-	-	-	-
Never smoker	31.44 (11.61–85.1)	**<** 0.001^***^	37.35 (12.42–135.21)	**<** 0.001^***^
Brain metastasis = yes (ref.: no)	2.59 (1.33–5.02)	0.005^**^	5.52 (2.22–14.69)	**<** 0.001^***^
Peritoneal metastasis = yes (ref.: no)	0 (–)	0.985	0 (–)	0.989
Bone metastasis = yes (ref.: no)	2.63 (1.33–5.19)	0.005^**^	2.94 (1.20–7.60)	0.020^*^

^*^p < 0.05, ^**^p < 0.005, ^***^p < 0.001. Significant results are in bold

*95% CI* 95% confidence interval, *OA* oncogenic alteration, *OR* odds ratio, *ref.* level of reference, *RFs* radiomics features, *nsOA* non-smoker oncogenic alteration, *sOA* smoker oncogenic alteration, *WHO-PS* World Health Organization performance status, *WT* wild-type

In univariable analysis, wild-type vs any OA yielded statistically significant differences for sex, pleural metastasis, cluster-C, and cluster-M. The same was true when comparing wild-type and sOA, owing to the large representation of patients with sOAs. The comparison of wild-type vs nsOA yielded other differences, such as the absence of tobacco addiction and high WHO-PS, brain or bone metastasis, and Euclidean lesion to centroid distances. Clusters C and M2 also highlighted differences. Finally, sex, tobacco addiction, brain and bone metastases also differentiated nsOAs from sOAs.

Multivariable analysis confirmed the influence of sex and differences between Cluster-M sub-clusters, smoking, and bone or brain metastases.

**Fig. 4:**
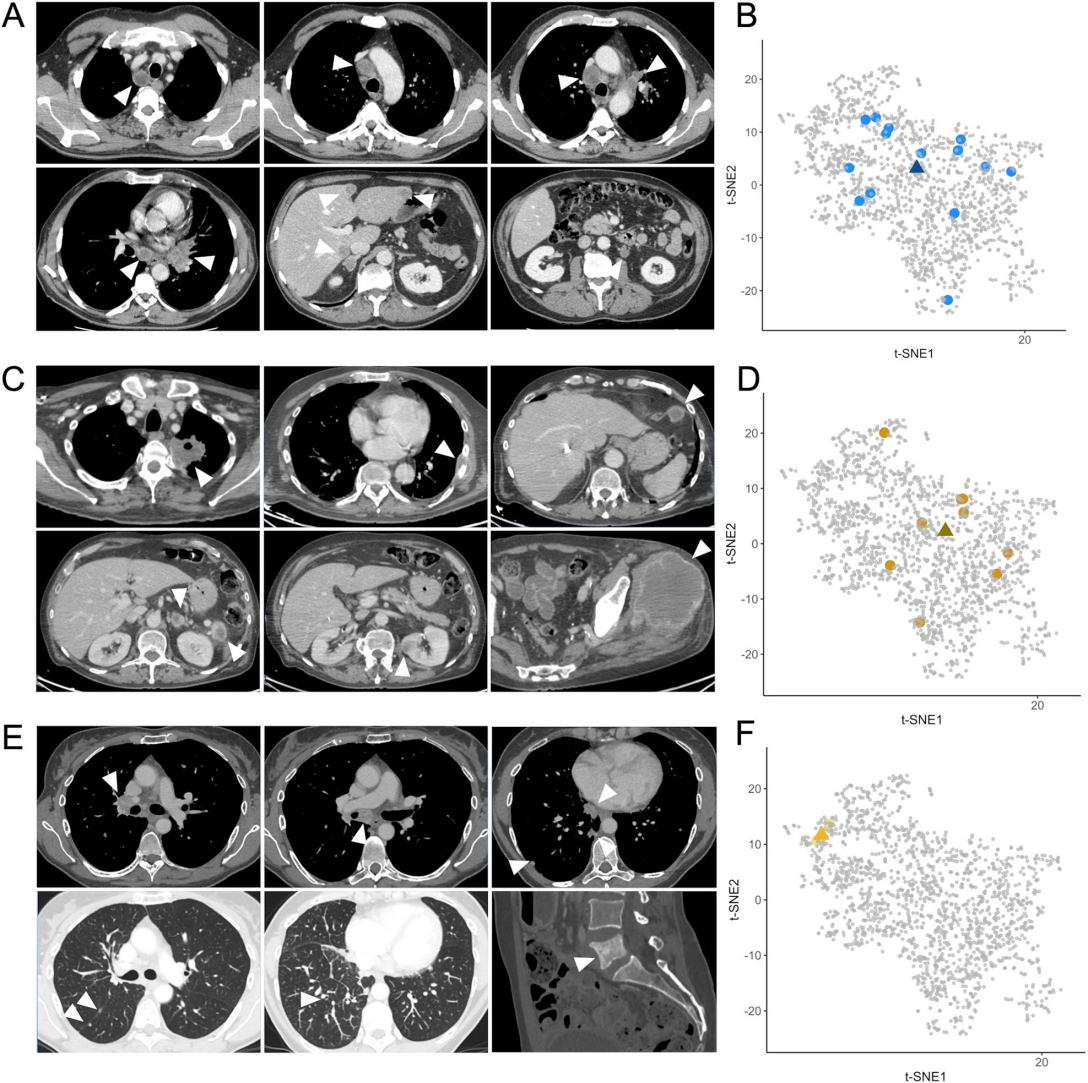
Representative CT scans and radiomics-based lesion dispersion in three patients. **A** Baseline CT of a 59-year-old man, a former smoker, with wild-type LUAD showing heterogeneous supra- and infra-diaphragmatic lymphadenopathies, a left hilar primary tumor, and adrenal and liver metastases (white arrowheads). **B** Lesion locations for this patient (light blue circles) on a 2D t-SNE projection of RFs, with the patient-specific centroid marked by a dark blue triangle. High lesion dispersion is illustrated by the spread around the centroid. Light gray dots represent lesions from the 360 other patients. **C** Baseline CT of another 59-year-old man, active smoker, with LUAD harboring *KRAS* G12D and *TP53* mutations, classified as having sOA. Imaging showed a left upper lobe primary tumor with bone, adrenal, renal, peritoneal, and soft-tissue metastases (white arrowheads). **D** Lesion distribution (gold circles) with a dark gold triangle representing the centroid shows moderately high lesion dispersion. **E** Baseline CT of a 55-year-old woman, never smoker, with *ALK*-rearranged LUAD (i.e., non-smoker OA). Imaging revealed a right lower lobe primary tumor, mediastinal lymphadenopathies, pleural metastases, lymphangitis, and lumbar sclerotic bone metastasis (white arrowheads). **F** Lesion locations (yellow circles) and centroid (dark yellow triangle) indicate minimal dispersion of the four segmented lesions, highlighting lesion similarity in the radiomic space. LUAD, lung adenocarcinoma; sOA, Smoker-related oncogenic alteration

Figure 4 shows representative CTs of wild-type, sOA, and nsOA patients and the lesion dispersions in a 2D-t-SNE representation based on their RFs, illustrating the shorter dispersion of lesion-to-centroid distance for the patient in the nsOA group, intermediate distances in the sOA patient, and broader distances in the wild-type patient.

### Added value of radiomics to clinical–radiological data to predict OA

Radiomics-based models consistently outperformed clinical–radiological models in discriminating oncogenic profiles across all subgroups in the 1000 out-of-bag test sets of the Monte Carlo cross-validation. Supplemental Table ST6 shows AUROCs in training and validation sets, comparing patients with a wild-type status and the three groups of at least one OA, nsOA, and sOA. Figure 3D–F shows AUROC comparing wild-type to respectively at least one OA, nsOAs, and sOAs in various combinations of factors, i.e., clinical–radiological variables, clusters, and Euclidean distances, again stressing the homogeneity of nsOAs. In the wild-type vs at least one OA comparison, integrating Cluster-M with sex and pleural metastasis increased the average AUROC from 0.593 (95% CI = 0.533–0.657) to 0.655 (95% CI = 0.591–0.718) (Fig. 3D). In the wild-type vs nsOA subgroup, the addition of Cluster-M and the range of Euclidean distances to a clinical model (including sex, tobacco use, WHO-PS, brain and bone metastases) improved the AUROC from 0.838 (95% CI = 0.749–0.916) to 0.849 (95% CI = 0.755–0.922) (Fig. 3E). Similarly, in the wild-type vs sOA analysis, combining pleural metastasis with Cluster-M raised the AUROC from 0.555 (95% CI = 0.505–0.603) to 0.637 (95% CI = 0.573–0.701) (Fig. 3F).

### Associations with patient outcome

**Fig. 5:**
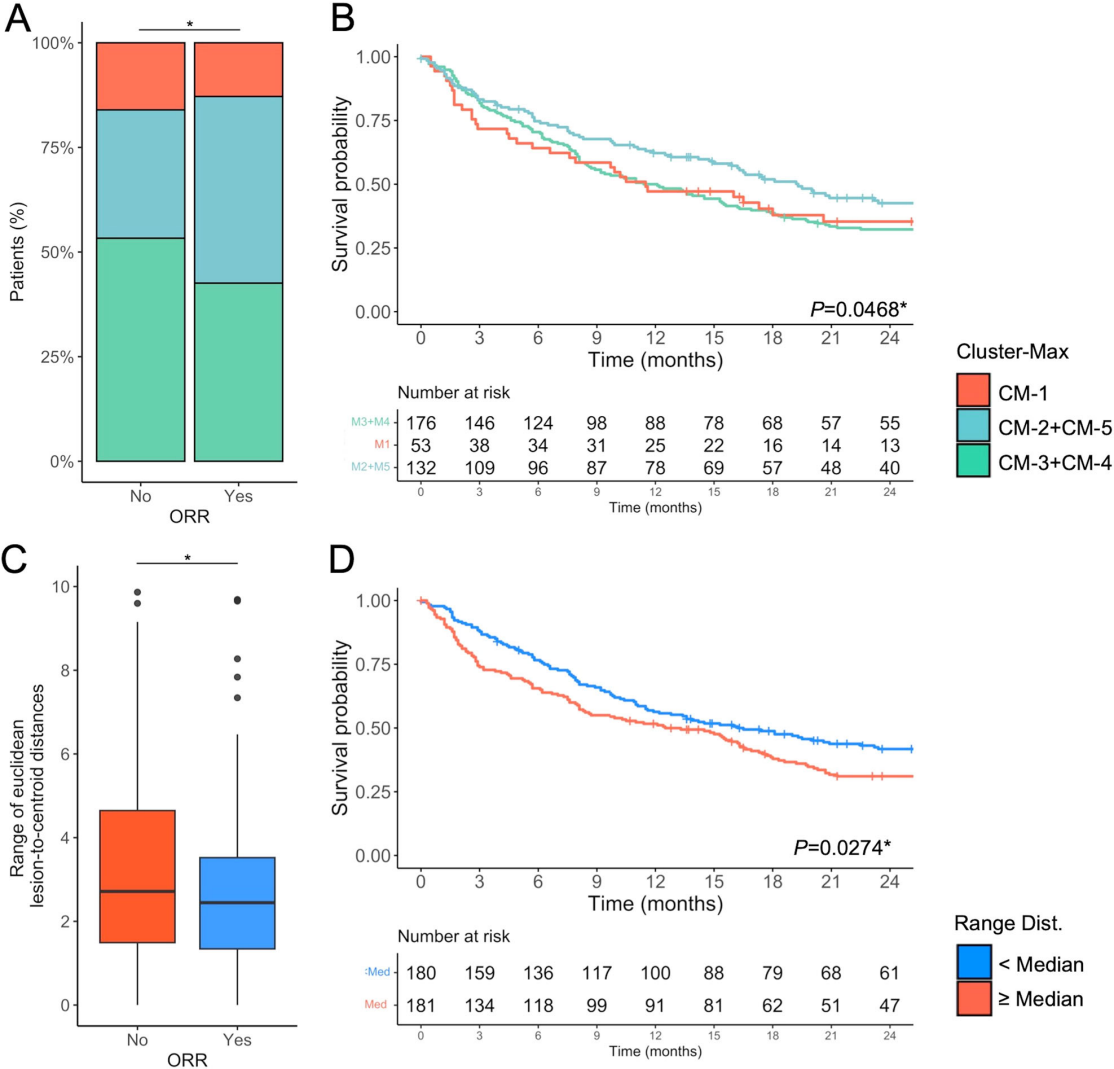
Associations between RFs and patient outcomes in the full cohort. **A** Distribution of Cluster-M groups in patients with vs without objective response (ORR) to first-line therapy. **B** Kaplan–Meier curves of OS stratified by Cluster-M group. **C** Boxplots showing the range of lesion-to-centroid Euclidean distances according to ORR. **D** Kaplan–Meier curves of OS based on high vs low radiophenotypic dispersion, dichotomized by the median range of Euclidean distances. ^*^p < 0.05

Supplemental Table ST7 shows how clustering was found to relate to ORR and outcome, graphically represented in Fig. 5.

Within Cluster-M, patients in sub-clusters M2 + M5 showed higher ORR (*p* = 0.011) and experienced longer OS (*p* = 0.016). A greater range of lesion-to-centroid Euclidean distance, reflecting higher intra-patient radiophenotypic dispersion, was linked to lower ORR *(p* = *0*.040) and shorter OS (*p* < 0.001). This association was held within the subgroup of patients treated with tyrosine kinase inhibitors (*n* = 46), where patients with higher lesion dispersion had a significantly poorer OS (*p* = 0.004). Subsequently, multivariable Cox regressions adjusting for age, sex, WHO-PS, and TNM stage, along with the variables significant in univariable analyses, were performed in both the entire cohort and the TKI-treated cohort (Supplementary Table ST8). The range of lesion-to-centroid Euclidean distances remained independently associated with OS in both analyses (entire cohort: HR = 1.07, 95% CI = 1.01–1.12, *p* = 0.0143; TKI cohort: HR = 1.33, 95% CI = 1.09–1.63, *p* = 0.0055)

## Discussion

This study aimed at assessing associations between unsupervised clustering of CT-based RFs and clinically relevant OAs, response rate, and OS on the basis of a large radiomic analysis of all lesions of patients with stage III/IVb metastatic LUAD.

Three clustering strategies were developed using RFs derived from a centroid-based integration of all lesions (Cluster-C), the largest lesion per patient (Cluster-M), and pre-sampling analysis of the biopsied lesion (Cluster-B) that was submitted to molecular analysis.

These radiophenotypes, identified without any *a priori* criterion, appeared to be strongly linked to underlying OAs. Cluster-M2 + M5 was significantly enriched in patients harboring *EGFR* mutations and was independently predictive of OA, sOA, and nsOA in multivariable analyses. This work differs from the prior study based on a subset of the same cohort [12], which used a supervised modeling strategy to predict OS. In contrast, the present study employs an unsupervised radiomic clustering framework to explore intrinsic imaging phenotypes and their associations with OAs and treatment response, thereby addressing distinct scientific objectives.

Cluster-M1 was significantly enriched in patients harboring *KRAS*, *TP53, PI3KCA,* and *MET* alterations, and was independently predictive of at least one OA and especially sOA. Cluster-C1, characterized by distinct CT-based radiomic patterns, showed strong associations with at least one OA and sOA in univariable analyses. By contrast, Cluster M3 + M4 was more prevalent in wild-type patients.

In agreement with the study by Perez-Johnston et al, these results highlight the capacity of radiomic profiling to distinguish molecularly distinct MLUAD subtypes [11]. Clustering on features from the largest lesion (Cluster-M) was shown to outperform centroid-based multi-site clustering (Cluster-C), demonstrating the limited incremental value of complex multi-site integration over single-site radiomics. The following hypotheses may explain this observation. Larger lesions typically exhibit greater radiomic heterogeneity and more stable feature estimates, which may enhance their ability to capture biologically relevant signals. Moreover, the largest lesion is often the one biopsied for molecular profiling; therefore, RFs from this lesion are more likely to reflect the genotypic reference standard, whereas smaller lesions (often not sampled) may introduce noise when aggregated.

Another finding of this study was that nsOA patients displayed higher intra-group radiomic consistency. Specifically, nsOA patients had significantly lower lesion-to-centroid Euclidean distance ranges compared to wild-type patients, suggesting a higher degree of radiophenotypic homogeneity across metastatic sites. This was further supported by ARI calculations, where nsOA patients exhibited the highest clustering concordance across methods, reinforcing the idea that nsOA molecular subtypes express more uniform imaging phenotypes. This result supports, at the multi-site level, the radiogenomic hypothesis that macroscopic radiologic heterogeneity reflects underlying molecular heterogeneity [6, 21]. Previous studies have shown that LUADs from non-smokers exhibit lower tumor mutational burden (< 4 mutations/Mb), whereas LUADs with *KRAS*, *BRAF*, or *MET* alterations, more common in smokers, display higher mutational burden (> 4 mutations/Mb) [22–24]. In the present metastatic cohort, this biological variability is mirrored radiologically, with greater lesion-to-lesion radiomic dissimilarity observed in patients with wild-type and sOA subtypes, consistent with higher molecular heterogeneity.

Furthermore, radiomics successfully differentiated OA subgroups from wild-type patients. Integrating Cluster-M and lesion dispersion provided additional discriminatory power. These improvements provide non-invasive biomarkers that could allow triage of patients prior to molecular screening or in case of failure of the latter. These results also add to those of Perez-Johnston et al, which did not evaluate the predictive value in a multivariable setting including clinical and radiological covariates and a resampling scheme such as Monte-Carlo cross-validation [11].

Single- and multi-site radiomics data also captured clinically relevant behaviors. Cluster-M2 + M5, linked to nsOA, appeared to be independently associated with a higher ORR and longer OS, in agreement with literature data [25]. This cluster also showed a greater proportion of women and was characterized by more frequent bone metastases and carcinomatous lymphangitis. These findings suggest a recognizable morphological profile that aligns with known nsOA biology. Together, the molecular, radiomic, and morphological concordance supports the biological validity of the clusters and indicates that the radiomics-derived groups reflect both micro-scale texture signatures and macro-scale imaging phenotypes. Conversely, high lesion-to-centroid dispersion, indicative of greater inter-lesional radiophenotypic heterogeneity, was found to be associated with lower ORR and significantly shorter OS. These associations were preserved in patients with at least one OA within the subgroup of patients treated with tyrosine kinase inhibitors, highlighting the potentially predictive value of radiomic heterogeneity, in the context of targeted therapy, consistent with literature data suggesting that tumors with greater spatial heterogeneity may harbor subclonal populations with differential therapeutic sensitivity [26].

From a translational standpoint, radiomics could thus support an early identification of patients likely to harbor actionable molecular alterations, accelerating the time-to-genotyping, guiding liquid biopsy use, and optimizing treatment selection. In clinical practice, radiomic clustering could help prioritize patients for urgent molecular testing, flag cases where biopsy is unsafe or inconclusive, or complement biopsy-based genotyping by providing whole-tumor burden information that captures spatial heterogeneity beyond a single sampled lesion. However, practical deployment remains limited by the need for robust automated segmentation, scanner-related variability, and the absence of harmonized acquisition protocols. Future studies should explore the added value of combining radiomics with baseline circulating tumor DNA to reduce the reliance on invasive tissue biopsies [27]. In parallel, fully automated deep-learning workflows may further enhance patient stratification by extracting personalized deep RFs and integrating information from multiple tumor lesions [28, 29].

A first limitation of this study is that it was retrospective and monocentric. Radiomics analysis was performed on CT scans acquired across multiple centers and CT systems, without standardized acquisition protocols, particularly regarding contrast administration. Brain lesions were imaged in a delayed post-contrast phase, although a prior study has shown that post-injection timing can significantly influence texture-based RFs [30]. In addition, we could not verify the quality of contrast injection or ensure full comparability of portal-venous phase acquisitions across scanners. Hence, these sources of heterogeneity may have impacted radiomic measurements. Although intensity discretization and rescaling were performed according to IBSI recommendations, no additional inter-scanner harmonization method, such as ComBat, was applied. Indeed, the study involved a large number of heterogeneous CT systems with incomplete acquisition metadata, which prevented the implementation of robust harmonization frameworks. This may have introduced residual scanner-related variability in the RFs. Furthermore, the heterogeneity of acquisition protocols and scanner types may introduce variability in RFs and radiomics clustering and slightly impair subsequent model performance. However, this variability also reflects real-world imaging conditions and may ultimately improve the generalizability of radiomics-based clustering approaches when applied to diverse clinical settings. Secondly, the radiomics pipeline required solid tumor lesions ≥ 1 cm³, unsuitable for patients with non-measurable disease, such as sclerotic bone metastases, meningeal or lymphangitic spread, or malignant effusions. Third, outcome analyses were exploratory and limited to univariate models due to sample size constraints, preventing multivariable adjustment. Fourth, genomic analyses focused on major OAs as recommended by current French guidelines, without stratifying by specific mutation subtypes or including emerging or less common molecular drivers. Fifth, we did not explicitly distinguish primary tumors from metastatic lesions when establishing the radiomic clusters, although these lesions may differ in morphology and biological behavior. This choice was intentional: in patients with lung metastases, it was often impossible to ascertain with certainty whether a segmentable lung lesion represented the true primary tumor. Explicitly separating primaries from metastases would therefore have introduced misclassification bias or led to the exclusion of a substantial proportion of patients, reducing statistical power. To balance these constraints, clustering was based on the largest lesion, which is also more likely to capture intra-tumoral heterogeneity, while all lesions were still included in the assessment of inter-site heterogeneity.

In conclusion, this study extends prior findings in stage I LUAD, demonstrating that CT-based radiomic clustering is associated with key OAs, response to treatment, and OS in stage IIIB–IV disease. Clustering based on the largest lesion tended to outperform multi-site approaches and showed strong associations with actionable OAs, both smoking- and non-smoking-related. Higher inter-lesion radiomic heterogeneity corresponded to greater molecular diversity, particularly in wild-type and smoker-associated tumors. These results support the potential of radiomics as a non-invasive tool to inform molecular testing, treatment decisions, and outcomes across all LUAD stages.

## Supplementary information

ELECTRONIC SUPPLEMENTARY MATERIAL

## Abbreviations

ARI: Adjusted Rand index
AUROC: Area under the curve
CI: Confidence interval
Cluster-B: Clustering result based on the radiomic features from the biopsied lesion
Cluster-C: Clustering result based on the radiomic features from the centroid lesions
Cluster-M: M for Maximum, i.e., clustering result based on the radiomic features from the largest lesion
HR: Hazard ratio
IQR: Interquartile range
LUAD: Lung adenocarcinoma
MLUAD: Metastatic lung adenocarcinoma
nsOA: Non-smoker-related oncogenic alteration
OA: Oncogenic alterations
ORR: Overall response rate
OS: Overall survival
PD-L1: Programmed-death ligand 1
RF: Radiomic feature
sOA: Smoker-related oncogenic alteration
WHO-PS: World Health Organization performance status
