# Supplementary material for: Single and multi-site CT-based radiogenomics analysis of metastatic lung adenocarcinoma and correlations with outcome
Source: Eur Radiol. 2026 Jan 16;36(6):5109–24. doi: 10.1007/s00330-025-12292-8 (PMC13212765; doi:10.1007/s00330-025-12292-8)
Supplement: Supplementary file 1 — ELECTRONIC SUPPLEMENTARY MATERIAL [file 330_2025_12292_MOESM1_ESM.pdf]

Single and Multi-Site CT-based radiogenomics analysis of metastatic lung adenocarcinoma and correlations with outcome

ELECTRONIC SUPPLEMENTARY MATERIAL

SUPPLEMENTAL METHOD SM1. Details of the radiomics pipeline

CT post-processing and segmentations

Contrast-enhanced CT covering in the abdominal kernel were pseudonymized and converted from the DICOM to the nifti format using the dcm2niix free converter (github.com/rordenlab/dcm2niix). CTs were then exported to the LIFEx freeware (v7.1.1, https://www.lifexsoft.org/), which adheres to the Imaging Biomarker Standardization Initiative (IBSI) guidelines [1-3]. All tumor lesions with a volume ≥ 1 cm^3 were manually segmented in 3D (without any assistance), slice-by-slice, by two radiologists (C.M.G, with 2 years of experience in oncologic imaging, and A.C. with 8 years of experience in oncologic imaging) within LIFEx. Segmentations performed by the youngest radiologist were systematically reviewed and confirmed by the senior radiologist. For bone metastases, only the extra-osseous soft tissue component was segmented, and for lung lesions, cavitated areas were excluded.

To assess inter-segmentation reproducibility, 100 randomly selected volume-of-interests were automatically eroded by one voxel to generate secondary volumes-of-interest for reproducibility analysis [4].

Additionally, when a segmented lesion corresponded to the biopsied tumor used for molecular profiling, this correspondence was recorded for downstream analyses.

Lesion-based RF extraction.

The image densities were discretized into 120 gray levels with a fixed bin width of 2.5 Hounsfield Units (HU), covering a range from -100 HU to +200 HU to capture the spectrum of soft-tissue densities relevant for radiomics.

Voxel dimensions were resampled to a uniform size of $1 \times 1 \times 2$ mm^3 using B-spline interpolation to ensure spatial standardization across scans.

A total of 121 radiomics features (RFs) were extracted in 3D, following the definitions and formulas provided on the LIFEx website (lifexsoft.org/index.php/resources/documentation) [1].

Gray-level co-occurrence matrix features were computed in 13 directions using a displacement of one voxel.

RF filtering and transformation.

RFs were recalculated for the 100 eroded volume-of-interest to assess their robustness to segmentation variability. Intra-class correlation coefficients (ICCs) were computed with the following arguments: model: ‘two way’, type: ‘agreement’, unit: ‘single’ (icc function from the ‘irr’ R package). Next, only features with an ICC > .85 were retained for subsequent analyses. Additionally, features with near-zero variance across the cohort were excluded to avoid redundancy and instability in modeling. As a result, 68 RFs were included in the final dataset, comprising 10

shape-based features, 26 histogram-based features, and 32 second-order texture features.
To ensure comparability and improve the reliability of statistical analyses, RFs were then centered, scaled, and normalized using the Yeo-Johnson transformation [5]. This normalization technique is suited for skewed and non-normally distributed data, as it can handle both positive and negative values while stabilizing variance and minimizing the influence of outliers on distance-based methods and multivariate modeling.

SUPPLEMENTAL METHOD SM2. Principle of consensus clustering algorithm and selection of cluster number

The ConsensusClusterPlus algorithm is widely-used for unsupervised class discovery, and particularly useful to identify robust clusters in high-dimensional data (such as gene expression or radiomics data).This data-driven approach, free from any a priori assumptions, aimed to identify a robust and meaningful number of clusters within the patient cohort [6]. The optimal number of clusters (k) was determined by evaluating clustering stability across multiple resampled subsets of the data. Specifically, clustering was performed with k ranging from 2 to 10, using 80% subsampling and 1000 iterations per k value. Cluster stability was assessed using several criteria provided by the ConsensusClusterPlus framework, including cumulative distribution of function plots, delta area plots (to evaluate incremental stability), and consensus heatmaps (to visually inspect separation and consistency across clusters).
Additionally, intra-cluster consensus scores were calculated by averaging consensus values among samples within each cluster. Higher intra-cluster consensus scores indicate more stable and homogeneous groupings. The final selection of k was based on maximizing clustering stability and ensuring clinical interpretability.

SUPPLEMENTAL METHOD SM3. Principle of Adjusted Rand Index (ARI)

ARI is a statistical measure that quantifies the degree of concordance between two partitions by considering all pairs of samples and counting those that are assigned consistently in both clustering (i.e., together in both or apart in both) (7). Unlike the original Rand index, ARI adjusts the score to account for the expected similarity of all pairwise assignments due to random chance, resulting in a value that typically ranges from −1 (no agreement) to 1 (perfect agreement), with 0 indicating random clustering agreement.

REFERENCES

1. Nioche C, Orlhac F, Boughdad S, et al. LIFEx: A Freeware for Radiomic Feature Calculation in Multimodality Imaging to Accelerate Advances in the Characterization of Tumor Heterogeneity. Cancer Res. 2018;78(16):4786–4789. doi: 10.1158/0008-5472.CAN-18-0125.
2. The image biomarker standardisation initiative — IBSI 0.0.1dev documentation. . https://ibsi.readthedocs.io/en/latest/. Accessed June 27, 2021.

3. Zwanenburg A, Vallières M, Abdalah MA, et al. The Image Biomarker Standardization Initiative: Standardized Quantitative Radiomics for High-Throughput Image-based Phenotyping. Radiology. 2020;295(2):328–338. doi: 10.1148/radiol.2020191145.
4. Lambin P, Leijenaar RTH, Deist TM, et al. Radiomics: the bridge between medical imaging and personalized medicine. Nat Rev Clin Oncol. 2017;14(12):749–762. doi: 10.1038/nrclinonc.2017.141.
5. Yeo I-K, Johnson RA. A New Family of Power Transformations to Improve Normality or Symmetry. Biometrika. [Oxford University Press, Biometrika Trust]; 2000;87(4):954–959.
6. Wilkerson MD, Hayes DN. ConsensusClusterPlus: a class discovery tool with confidence assessments and item tracking. Bioinforma Oxf Engl. 2010;26(12):1572–1573. doi: 10.1093/bioinformatics/btq170.
7. Warrens MJ, van der Hoef H. Understanding the Adjusted Rand Index and Other Partition Comparison Indices Based on Counting Object Pairs. J Classif. 2022;39:487–509. doi: 10.1007/s00357-022-09413-z.

SUPPLEMENTAL METHOD SM4. Packages and software used in the study.
All resources used in the study are free and open-access.

Packages & softwares	version	Function	Use	Available at
Dicom2niix	v1.0.20211006	dcm2niix	Converting DICOM to nifti format	https://github.com/rordenlab/dcm2niix
LIFEx	v7.1.17	-	Processing imaging and extracting radiomics features	https://www.lifexsoft.org/
Rstudio	v1.4.1106	-	Scripting in R	https://posit.co/download/rstudio-desktop/
R	v4.1.0	-	Programing language for statistical analyses	https://www.r-project.org/
dplyr	v1.1.2	left_join, filter, select, summarize, pull, mutate	Manipulating data	https://github.com/tidyverse/dplyr
purrr	v0.3.4	map, map2, pmap	Applying functions iteratively on various numbers of inputs	https://github.com/tidyverse/purrr
openxlsx	v4.2.4	read.xlsx, write.xlsx	Opening and saving .xslx data	https://github.com/ycphs/openxlsx
lubridate	v1.7.10	dmy, durations	Manipulating dates and calculating durations	https://github.com/tidyverse/lubridate
irr	v0.84.1	icc	Calculating intra-class correlation coefficient	https://github.com/cran/irr
car	v3.0-10	powerTransform	Performing the Yeo-Johnson transformation	https://cran.r-project.org/web/packages/car/index.html
ConsensusClusterPlus	v1.58.0	ConsensusClusterPlus	Performing consensus clustering plus algorithm	https://bioconductor.org/packages/release/bioc/html/ConsensusClusterPlus.html
mclust	v6.1	adjustedRandIndex	Computing the adjusted Rand index to compare two classifications	https://cran.r-project.org/web/packages/mclust/index.html
MASS	v7.3-54	stepAIC	Performing stepwise analysis	https://github.com/cran/MASS
pROC	v1.17.0.1	roc, ci.auc, auc	Calculating area under the ROC curve	https://cran.r-project.org/web/packages/pROC/index.html
survival	v3.5-8	survdiff, survfit, Surv, coxph	Performing survival analysis (survival rates, median survival, log-rank test, Cox regression)	https://cran.r-project.org/web/packages/survival/index.html
caret	v6.0-88	nzv	Identifying features with near zero variance	https://topepo.github.io/caret/
ggplot2	v3.4.2	ggplot, ggsave	Drawing plots and saving them	https://github.com/tidyverse/ggplot2
cowplot	v1.1.1	plot_grid	Creating composed plots made of multiple individual plots	https://cran.r-project.org/web/packages/corrplot/index.html
Rtsne	v0.16	Rtsne	Dimensionality reduction	https://cran.r-project.org/web/packages/Rtsne/index.html
RColorBrewer	v1.1-2	brewer.pal	Creating color palettes	https://cran.r-project.org/web/packages/RColorBrewer/index.html

survminer	v0.4.9	ggsurvplot	creating Kaplan-Meier curves	https://github.com/kassambara/survminer
base	v4.6.0	scale, median, min, max, quantile, mean, replicate	Basic descriptive statistics, creating samples for Monte-Carlo cross validation	https://github.com/cran/base
stats	v3.6.2	dist, shapiro.test, wilcox.test, chisq.test, kruskal.test, relevel, glm	Basic inferential statistics including binary logistic regression	https://github.com/cran/stats

SUPPLEMENTAL METHOD SM5. Checklist for Artificial Intelligence in Medical Imaging (CLAIM, 2024 version) checklist.

Section / Topic	No.	Item	Page / Line	No	NA
TITLE / ABSTRACT					
	**1**	Identification as a study of AI methodology, specifying the category of technology used (e.g., deep learning)	**1/4**		
ABSTRACT					
	**2**	Summary of study design, methods, results, and conclusions	**1/25-45**		
INTRODUCTION					
	**3**	Scientific and/or clinical background, including the intended use and role of the AI approach	**4/2-56**		
	**4**	Study aims, objectives, and hypotheses	**4-5/57-3**		
METHODS					
Study Design	**5**	Prospective or retrospective study	**5/11**		
	**6**	Study goal	**4-5/57-3**		
Data	**7**	Data sources	**5/15-27**		
	**8**	Inclusion and exclusion criteria	**5/15-27**		
	**9**	Data pre-processing	**SM1**		
	**10**	Selection of data subsets			**x**
	**11**	De-identification methods			**x**
	**12**	How missing data were handled	**8/29-31**		
	**13**	Image acquisition protocol	**6/22-36**		
Reference Standard	**14**	Definition of method(s) used to obtain reference standard	**6/37-49**		
	**15**	Rationale for choosing the reference standard			**x**
	**16**	Source of reference standard annotations	**8/20-31**		
	**17**	Annotation of test set			**x**
	**18**	Measures of inter- and intra-rater variability of features described by the annotators	**SM1**		
Data Partitions	**19**	How data were assigned to partitions	**8/20-31**		
	**20**	Level at which partitions are disjoint			**x**
Testing Data	**21**	Intended sample size			**x**

Section / Topic	No.	Item	Page / Line	No	NA
Model	**22**	Detailed description of model	**8/17-21**		
	**23**	Software libraries, frameworks, and packages	**SM4**		
	**24**	Initialization of model parameters			**x**
Training	**25**	Details of training approach	**SM2**		
	**26**	Method of selecting the final model	**SM2**		
	**27**	Ensembling techniques			**x**
Evaluation	**28**	Metrics of model performance	**8/11-31**		
	**29**	Statistical measures of significance and uncertainty	**8/11-31**		
	**30**	Robustness or sensitivity analysis	**SM1**		
	**31**	Methods for explainability or interpretability		**x**	
	**32**	Evaluation on internal data			**x**
	**33**	Testing on external data			**x**
	**34**	Clinical trial registration			**x**
RESULTS					
Data	**35**	Numbers of patients or examinations included and excluded	**Fig. 1**		
	**36**	Demographic and clinical characteristics of cases in each partition	**8-9/44-13**		
Model performance	**37**	Performance metrics and measures of statistical uncertainty	**10-11/42-11**		
	**38**	Estimates of diagnostic performance and their precision	**10-11/42-11**		
	**39**	Failure analysis of incorrect results		**x**	
DISCUSSION					
	**40**	Study limitations	**13-14/47-2**		
	**41**	Implications for practice, including intended use and/or clinical role	**13/31-45**		
OTHER INFORMATION					
	**42**	Provide a reference to the full study protocol or to additional technical details			**x**
	**43**	Statement about the availability of software, trained model, and/or data	**8/33-36**		
	**44**	Sources of funding and other support; role of funders			**x**

* Indicate page and/or line number for each checklist item that is present. NA = not applicable.
Tejani AS, Klontzas ME, Gatti AA, et al. Checklist for Artificial Intelligence in Medical Imaging (CLAIM):2024 Update. Radiol Artif Intell 2024;6(4):e240300. https://doi.org/10.1148/ryai.240300

SUPPLEMENTAL TABLE ST1. Characteristics of segmented tumor lesions used for radiomics analyses.

Characteristics	Lesions
**No. per patient**	4 [3-6] (2-19)
**Lesion location**	
Adrenal	124/1721 (7.2)
Bone	73/1721 (4.2)
Brain	70/1721 (4.1)
Peritoneal carcinomatosis	45/1721 (2.6)
Liver	141/1721 (8.2)
Infra-diaphragmatic lymph node	47/1721 (2.7)
Supra-diaphragmatic lymph node	722/1721 (42)
Lung	394/1721 (22.9)
Ovary	1/1721 (0.1)
Pancreas	7/1721 (0.4)
Pericardium	1/1721 (0.1)
Pleura	35/1721 (2)
Spleen	16/1721 (0.9)
Kidney	6/1721 (0.3)
Soft tissue	39/1721 (2.3)
**Primitive tumor**	307/1721 (17.8)
**Lesion longest diameter, (mm)**	23 [17-35] (10-156)
**Lesion volume, (cm^{3})**	57.9 [26.4-106.6] (1-860.4)
**Sum of segmented lesion volumes per patient, (cm^3)**	58.2 [27-102.7] (2.1-870)
**Clearly identified biopsied lesions with matched RFs**	180/1721 (10.5)
**Locations of the biopsied lesions with matched RFs**	
Adrenal	7/180 (3.9)
Bone	18/180 (10)
Brain	4/180 (2.2)
Peritoneal carcinomatosis	3/180 (1.7)
Kidney	1/180 (0.6)
Liver	8/180 (4.4)
Lymph node	23/180 (12.8)
Primitive lung cancer	110/180 (61.1)
Pleura	2/180 (1.1)
Soft tissue	4/180 (2.2)

NOTE - Data are numbers of lesions with percentages in parentheses for categorical variables, and median, interquartile range and minimum-maximum range for numeric variables. Abbreviations:No.: number, RFs: radiomic features.

SUPPLEMENTAL TABLE ST2. Adjusted Rand Indices to assess the similarity between the 3 clustering (Cluster-C, Cluster-M and Cluster-B) in the entire cohort and depending on mutational status.

Cohort	Adjusted Rand Index		
	Cluster-B vs Cluster-C	Cluster-B vs Cluster-M	Cluster-M vs Cluster-C
**All patients**	0.236	0.330	0.417
**sOA**	0.181	0.326	0.398
**nsOA**	**0.490**	**0.407**	**0.535**
**Wild-type**	0.388	0.291	0.530

NOTE.- The highest value (reflecting greatest similarity) for each comparison is emphasized in bold. Abbreviations: nsOA: non-smoker oncogenic alteration, sOA: smoker oncogenic alteration.

SUPPLEMENTAL TABLE ST3. Characteristics associated with Cluster-C.

Characteristics	Clustering based on the centroid RFs			
	Cluster-C1 (N=176)	Cluster-C2 (N=133)	Cluster-C3 (N=52)	*P*-value
**Age, years**[IQR](range)	64 [55.8-70.3] (22-83.20)	62.1 [55.1-68.1] (25-87.9)	65.4 [58.6-71.3] (42.7-83.2)	0.134
**Sex (Women)**	76/176 (43.2)	40/133 (30.1)	**33/52 (63.5)**	**<.001*** **
**Tabacco addiction**				.648
Never smoker	18/176 (10.2)	17/130 (13.1)	6/51 (11.8)	
Active smoker	78/176 (44.3)	62/130 (47.7)	27/51 (52.9)	
Past smoker	80/176 (45.5)	51/130 (39.2)	18/51 (35.3)	
**WHO PS**				.457
0	40/176 (22.7)	43/133 (32.3)	14/52 (26.9)	
1	98/176 (55.7)	64/133 (48.1)	28/52 (53.8)	
≥ 2	38/176 (21.6)	26/133 (19.5)	10/52 (19.2)	
**Staging**				**.043***
III-Iva	49/176 (27.8)	21/133 (15.8)	12/52 (23.1)	
IVb	127/176 (72.2)	**112/133 (84.2)**	40/52 (76.9)	
**No. of distinct metastatic organs**				.535
1	32/176 (18.2)	26/133 (19.5)	15/52 (28.8)	
2	46/176 (26.1)	37/133 (27.8)	13/52 (25)	
≥ 3	98/176 (55.7)	70/133 (52.6)	24/52 (46.2)	
**Metastasis locations on initial imaging**				
Lung metastasis	75/176 (42.6)	62/133 (46.6)	17/52 (32.7)	.227
Leptomeningeal carcinomatosis	3/176 (1.7)	0/133 (0)	0/52 (0)	.204
Epiduritis	12/176 (6.8)	7/133 (5.3)	**11/52 (21.2)**	**.001****
Brain metastasis	56/176 (31.8)	43/133 (32.3)	12/52 (23.1)	.430
Pericardial metastasis	6/176 (3.4)	9/133 (6.8)	1/52 (1.9)	.232
Miliary	4/176 (2.3)	5/133 (3.8)	1/52 (1.9)	.676
Carcinomatous lymphangitis	**32/176 (18.2)**	11/133 (8.3)	4/52 (7.7)	**.017***
Pleural metastasis	41/176 (23.3)	21/133 (15.8)	8/52 (15.4)	.187
Mucle metastasis	22/176 (12.5)	14/133 (10.5)	4/52 (7.7)	.604
Spleen metastasis	9/176 (5.1)	2/133 (1.5)	1/52 (1.9)	.179
Kidney metastasis	8/176 (4.5)	3/133 (2.3)	1/52 (1.9)	.448
Pancreatic metastasis	6/176 (3.4)	3/133 (2.3)	1/52 (1.9)	.765
N3 or N4 lymphadenopathy	108/176 (61.4)	89/133 (66.9)	31/52 (59.6)	.514
Liver metastasis	41/176 (23.3)	23/133 (17.3)	13/52 (25)	.347
Adrenal metastasis	68/176 (38.6)	37/133 (27.8)	15/52 (28.8)	.104
Subcutaneous metastasis	14/176 (8)	9/133 (6.8)	3/52 (5.8)	.841
Peritoneal carcinomatosis	21/176 (11.9)	11/133 (8.3)	1/52 (1.9)	.081
Bone metastasis	90/176 (51.1)	53/133 (39.8)	29/52 (55.8)	.065
**PD-L1 TPS**				.385
0%	66/175 (37.7)	48/124 (38.7)	21/44 (47.7)	
1-49%	55/175 (31.4)	32/124 (25.8)	14/44 (31.8)	

50-100%	54/175 (30.9)	44/124 (35.5)	9/44 (20.5)	
**Gene alterations at initial screening**				
ROS1	2/176 (1.1)	1/133 (0.8)	0/52 (0)	.724
HER2	3/176 (1.7)	5/133 (3.8)	1/52 (1.9)	.497
ALK	4/176 (2.3)	3/133 (2.3)	0/52 (0)	.548
KRAS	76/176 (43.2)	46/133 (34.6)	23/52 (44.2)	.253
TP53	**66/176 (37.5)**	14/133 (10.5)	8/52 (15.4)	**<.001*** **
EGFR	21/176 (11.9)	9/133 (6.8)	9/52 (17.3)	.092
BRAF	10/176 (5.7)	4/133 (3)	4/52 (7.7)	.353
MET	4/176 (2.3)	2/133 (1.5)	0/52 (0)	.522
POLE	0/176 (0)	0/133 (0)	0/52 (0)	-
STK11	17/176 (9.7)	10/133 (7.5)	1/52 (1.9)	.185
PI3KCA	6/176 (3.4)	3/133 (2.3)	1/52 (1.9)	.765

NOTE.- Data are number of patients with percentage in parentheses, except for numeric variables given as median, 1st and 3rd quartile range and minimum-maximum range. *: *P*<.05, **: *P*<.005, ***: *P*<.001. Significant results are in bold. Abbreviations: no.: number, tumor positive score, WHO-PS: World health organization performans status.

SUPPLEMENTAL TABLE ST4. Characteristics associated with Cluster-M.

Characteristics	Clustering based on the largest lesion RFs (Cluster-M)			
	Cluster-M1 (N=53)	Cluster-M2+M5 (N=132)	Cluster-M3+M4 (N=176)	*P*-value
**Age (years)**	64 [55.7-72.1] (42.5-83)	64.3 [55.9-70.2] (22-83.2)	62.2 [56.3-68.8] (25-87.9)	.423
**Sex (Women)**	15/53 (28.3)	**70/132 (53)**	64/176 (36.4)	**.001****
**Tabacco addition**				.463
Never smoker	3/53 (5.7)	17/132 (12.9)	21/172 (12.2)	
Active smoker	23/53 (43.4)	60/132 (45.5)	84/172 (48.8)	
Past smoker	27/53 (50.9)	55/132 (41.7)	67/172 (39)	
**WHO PS**				.695
0	12/53 (22.6)	32/132 (24.2)	53/176 (30.1)	
1	29/53 (54.7)	74/132 (56.1)	87/176 (49.4)	
≥ 2	12/53 (22.6)	26/132 (19.7)	36/176 (20.5)	
**Staging**				**.048***
III-IVa	18/53 (34)	32/132 (24.2)	32/176 (18.2)	
IVb	35/53 (66)	100/132 (75.8)	**144/176 (81.8)**	
**No. of distinct metastatic organs**				.713
1	12/53 (22.6)	22/132 (16.7)	39/176 (22.2)	
2	14/53 (26.4)	34/132 (25.8)	48/176 (27.3)	
≥ 3	27/53 (50.9)	76/132 (57.6)	89/176 (50.6)	
**Metastasis locations on initial imaging**				
Lung	21/53 (39.6)	56/132 (42.4)	77/176 (43.8)	.866
Leptomeningeal carcinomatosis	1/53 (1.9)	2/132 (1.5)	0/176 (0)	.230
Epiduritis	4/53 (7.5)	12/132 (9.1)	14/176 (8)	.916
Brain	17/53 (32.1)	39/132 (29.5)	55/176 (31.2)	.926
Pericardial	1/53 (1.9)	4/132 (3)	11/176 (6.2)	.247
Miliary	1/53 (1.9)	4/132 (3)	11/176 (6.2)	.247
Carcinomatous lymphangitis	7/53 (13.2)	**26/132 (19.7)**	14/176 (8)	**.010***
Pleura	14/53 (26.4)	29/132 (22)	27/176 (15.3)	.130
Mucle	9/53 (17)	13/132 (9.8)	18/176 (10.2)	.332
Spleen	2/53 (3.8)	5/132 (3.8)	5/176 (2.8)	.883
Kidney	2/53 (3.8)	7/132 (5.3)	3/176 (1.7)	.215
Pancreatic	4/53 (7.5)	2/132 (1.5)	4/176 (2.3)	.066
N3 or N4 lymphadenopathy	31/53 (58.5)	83/132 (62.9)	114/176 (64.8)	.705
Liver	12/53 (22.6)	28/132 (21.2)	37/176 (21)	.968
Adrenal	23/53 (43.4)	46/132 (34.8)	51/176 (29)	.131
Subcutaneous	5/53 (9.4)	11/132 (8.3)	10/176 (5.7)	.534
Peritoneal carcinomatosis	9/53 (17)	12/132 (9.1)	12/176 (6.8)	.079
Bone	21/53 (39.6)	**78/132 (59.1)**	73/176 (41.5)	**.004****
**PD-L1 TPS**				.233
0%	16/53 (30.2)	49/129 (38)	70/161 (43.5)	
1-49%	18/53 (34)	44/129 (34.1)	39/161 (24.2)	

50-100%	19/53 (35.8)	36/129 (27.9)	52/161 (32.3)	
**Gene alterations at initial screening**				
ROS1	1/53 (1.9)	1/132 (0.8)	1/176 (0.6)	.646
HER2	0/53 (0)	4/132 (3)	5/176 (2.8)	.449
ALK	1/53 (1.9)	3/132 (2.3)	3/176 (1.7)	.938
KRAS	**29/53 (54.7)**	53/132 (40.2)	63/176 (35.8)	**.048***
TP53	**22/53 (41.5)**	37/132 (28)	29/176 (16.5)	**<.001*****
EGFR	2/53 (3.8)	**22/132 (16.7)**	15/176 (8.5)	**.015***
BRAF	3/53 (5.7)	9/132 (6.8)	6/176 (3.4)	.385
MET	3/53 (5.7)	1/132 (0.8)	2/176 (1.1)	**.046***
POLE	0/53 (0)	0/132 (0)	0/176 (0)	-
STK11	5/53 (9.4)	11/132 (8.3)	12/176 (6.8)	.784
PI3KCA	**6/53 (11.3)**	1/132 (0.8)	3/176 (1.7)	**<.001*****

NOTE.- Data are number of patients with percentage in parentheses, except for numeric variables given as median, 1st and 3rd quartile range and minimum-maximum range. *: *P*<.05, **: *P*<.005, ***: *P*<.001. Significant results are in bold. Abbreviations: no.: number, tumor positive score, WHO-PS: World health organization performans status.

SUPPLEMENTAL TABLE ST5. Characteristics associated with Cluster-B.

Characteristics	Clustering based on the biopsied lesion (Cluster-B)						
	**Cluster-B1**	**Cluster-B2**	**Cluster-B3**	**Cluster-B4**	**Cluster-B5**	**Cluster-B6**	**P-value**
**Age (years)**	63.5 [59.6-67.5] (42.5-87.9)	62.1 [56.3-68.6] (42.7-80.4)	64 [60.5-70.4] (42.1-83.20)	63.6 [55.7-71.3] (22-80.3)	56.5 [51.6-70] (49-73.7)	64.5 [57.4-72.1] (44.6-79.7)	.857
**Sex (Women)**	10/19 (52.6)	6/26 (23.1)	30/59 (50.8)	12/36 (33.3)	**4/7 (57.1)**	7/33 (21.2)	**.018***
**Tabacco addiction**							.142
Never smoker	2/19 (10.5)	6/25 (24)	10/59 (16.9)	1/36 (2.8)	0/7 (0)	1/33 (3)	
Active smoker	8/19 (42.1)	11/25 (44)	20/59 (33.9)	20/36 (55.6)	4/7 (57.1)	16/33 (48.5)	
former smoker	9/19 (47.4)	8/25 (32)	29/59 (49.2)	15/36 (41.7)	3/7 (42.9)	16/33 (48.5)	
**WHO PS**							.104
0	8/19 (42.1)	5/26 (19.2)	13/59 (22)	8/36 (22.2)	0/7 (0)	11/33 (33.3)	
1	7/19 (36.8)	19/26 (73.1)	37/59 (62.7)	20/36 (55.6)	4/7 (57.1)	13/33 (39.4)	
≥ 2	4/19 (21.1)	2/26 (7.7)	9/59 (15.3)	8/36 (22.2)	3/7 (42.9)	9/33 (27.3)	
**Staging**							.069
III-IVa	4/19 (21.1)	1/26 (3.8)	15/59 (25.4)	10/36 (27.8)	2/7 (28.6)	13/33 (39.4)	
IVb	15/19 (78.9)	25/26 (96.2)	44/59 (74.6)	26/36 (72.2)	5/7 (71.4)	20/33 (60.6)	
**No. of distinct metastatic organs**							.876
1	6/19 (31.6)	6/26 (23.1)	10/59 (16.9)	9/36 (25)	1/7 (14.3)	9/33 (27.3)	
2	2/19 (10.5)	8/26 (30.8)	15/59 (25.4)	7/36 (19.4)	2/7 (28.6)	8/33 (24.2)	
≥ 3	11/19 (57.9)	12/26 (46.2)	34/59 (57.6)	20/36 (55.6)	4/7 (57.1)	16/33 (48.5)	
**Metastasis locations on initial imaging**							
Lung	10/19 (52.6)	8/26 (30.8)	27/59 (45.8)	14/36 (38.9)	5/7 (71.4)	11/33 (33.3)	.285
Leptomeningeal carcinomatosis	0/19 (0)	0/26 (0)	0/59 (0)	1/36 (2.8)	**1/7 (14.3)**	0/33 (0)	**.019***
Epiduritis	2/19 (10.5)	1/26 (3.8)	7/59 (11.9)	2/36 (5.6)	0/7 (0)	1/33 (3)	.520
Brain	5/19 (26.3)	8/26 (30.8)	14/59 (23.7)	12/36 (33.3)	5/7 (71.4)	7/33 (21.2)	.136
Pericardial	2/19 (10.5)	2/26 (7.7)	2/59 (3.4)	2/36 (5.6)	0/7 (0)	1/33 (3)	.761
Miliary	0/19 (0)	1/26 (3.8)	2/59 (3.4)	0/36 (0)	0/7 (0)	1/33 (3)	.822
Carcinomatous lymphangitis	2/19 (10.5)	1/26 (3.8)	11/59 (18.6)	7/36 (19.4)	2/7 (28.6)	5/33 (15.2)	.437
Pleural	4/19 (21.1)	4/26 (15.4)	14/59 (23.7)	8/36 (22.2)	1/7 (14.3)	10/33 (30.3)	.817

Mucle	3/19 (15.8)	3/26 (11.5)	4/59 (6.8)	3/36 (8.3)	1/7 (14.3)	6/33 (18.2)	.613
Spleen	2/19 (10.5)	0/26 (0)	3/59 (5.1)	1/36 (2.8)	0/7 (0)	3/33 (9.1)	.480
Kidney	1/19 (5.3)	0/26 (0)	3/59 (5.1)	1/36 (2.8)	1/7 (14.3)	2/33 (6.1)	.649
Pancreas	0/19 (0)	0/26 (0)	1/59 (1.7)	3/36 (8.3)	0/7 (0)	2/33 (6.1)	.322
N3 or N4 lymphadenopathy	11/19 (57.9)	17/26 (65.4)	34/59 (57.6)	23/36 (63.9)	4/7 (57.1)	19/33 (57.6)	.976
Liver	4/19 (21.1)	4/26 (15.4)	13/59 (22)	7/36 (19.4)	2/7 (28.6)	9/33 (27.3)	.910
Adrenal	7/19 (36.8)	8/26 (30.8)	19/59 (32.2)	18/36 (50)	3/7 (42.9)	13/33 (39.4)	.589
Subcutaneous	3/19 (15.8)	1/26 (3.8)	2/59 (3.4)	3/36 (8.3)	1/7 (14.3)	2/33 (6.1)	.450
Peritoneal carcinomatosis	3/19 (15.8)	1/26 (3.8)	6/59 (10.2)	3/36 (8.3)	2/7 (28.6)	5/33 (15.2)	.434
Bone	6/19 (31.6)	11/26 (42.3)	32/59 (54.2)	18/36 (50)	6/7 (85.7)	14/33 (42.4)	.170
**PD-L1 TPS score**							.393
0%	6/19 (31.6)	12/24 (50)	17/59 (28.8)	12/36 (33.3)	4/7 (57.1)	12/33 (36.4)	
1-49%	7/19 (36.8)	6/24 (25)	23/59 (39)	8/36 (22.2)	3/7 (42.9)	9/33 (27.3)	
50-100%	6/19 (31.6)	6/24 (25)	19/59 (32.2)	16/36 (44.4)	0/7 (0)	12/33 (36.4)	
**Gene alterations at initial screening**							
ROS1	0/19 (0)	0/26 (0)	2/59 (3.4)	0/36 (0)	0/7 (0)	0/33 (0)	.528
HER2	2/19 (10.5)	0/26 (0)	1/59 (1.7)	0/36 (0)	0/7 (0)	0/33 (0)	.055
ALK	0/19 (0)	1/26 (3.8)	1/59 (1.7)	1/36 (2.8)	0/7 (0)	1/33 (3)	.951
KRAS	10/19 (52.6)	6/26 (23.1)	21/59 (35.6)	20/36 (55.6)	3/7 (42.9)	17/33 (51.5)	.089
TP53	5/19 (26.3)	3/26 (11.5)	19/59 (32.2)	14/36 (38.9)	2/7 (28.6)	**17/33 (51.5)**	**.042***
EGFR	1/19 (5.3)	4/26 (15.4)	11/59 (18.6)	1/36 (2.8)	0/7 (0)	1/33 (3)	.054
BRAF	0/19 (0)	1/26 (3.8)	4/59 (6.8)	3/36 (8.3)	0/7 (0)	2/33 (6.1)	.793
MET	0/19 (0)	0/26 (0)	0/59 (0)	0/36 (0)	0/7 (0)	**3/33 (9.1)**	**.018***
POLE	0/19 (0)	0/26 (0)	0/59 (0)	0/36 (0)	0/7 (0)	0/33 (0)	-
STK11	1/19 (5.3)	1/26 (3.8)	6/59 (10.2)	5/36 (13.9)	0/7 (0)	2/33 (6.1)	.624
PI3KCA	0/19 (0)	1/26 (3.8)	1/59 (1.7)	1/36 (2.8)	0/7 (0)	4/33 (12.1)	.161

NOTE.- Data are number of patients with percentage in parentheses, except for numeric variables given as median, 1st and 3rd quartile range and minimum-maximum range. *: *P*<.05, **: *P*<.005, ***: *P*<.001. Significant results are in bold. Abbreviations: no.: number, tumor positive score, WHO-PS: World health organization performans status.

SUPPLEMENTAL TABLE ST6. Diagnostic performances of binary logistic regressions in the training and independent validation sets. Performance metrics was the area under the ROC curve (AUC), averaged over the 1000 random samplings of the study. Three binary classification tasks were achieved (left column) discriminating (i) wild-type (WT) patients vs. patients with at least one oncogenic alteration (OA), (ii) WT vs. nsOA, and (iii) WT vs. sOA.

Classification Tasks	Models (input variables)§	AUCs in Training set	AUCs in Validation set
**WT versus Any AO**	CR	0.604 (0.563-0.643)	0.593 (0.533-0.657)
	CR + Cluster-C	0.670 (0.629-0.711)	0.641 (0.574-0.707)
	CR + Cluster-M	0.679 (0.638-0.719)	**0.655 (0.591-0.718)**
	CR + Dist.	0.613 (0.570-0.655)	0.587 (0.517-0.655)
	CR + Cluster-C + Cluster-M	0.689 (0.647-0.73)	0.643 (0.574-0.706)
	CR + Cluster-M + Dist	0.683 (0.642-0.725)	0.648 (0.583-0.714)
	CR + Cluster-C + Dist	0.674 (0.633-0.717)	0.633 (0.565-0.699)
	CR + Cluster-C + Cluster-M + Dist	**0.692 (0.650-0.735)**	0.636 (0.570-0.701)
**WT versus nsAO**	CR	0.885 (0.844-0.928)	0.838 (0.749-0.916)
	CR + Cluster-C	0.904 (0.864-0.945)	0.843 (0.759-0.916)
	CR + Cluster-M	0.907 (0.868-0.946)	0.848 (0.746-0.921)
	CR + Dist.	0.900 (0.861-0.940)	0.846 (0.762-0.917)
	CR + Cluster-C + Cluster-M	0.914 (0.875-0.952)	0.837 (0.733-0.914)
	CR + Cluster-M + Dist	0.922 (0.886-0.956)	**0.849 (0.755-0.922)**
	CR + Cluster-C + Dist	0.917 (0.879-0.954)	0.847 (0.761-0.919)
	CR + Cluster-C + Cluster-M + Dist	**0.928 (0.893-0.966)**	0.841 (0.741-0.917)
**WT versus sAO**	CR	0.555 (0.524-0.585)	0.555 (0.505-0.603)
	CR + Cluster-C	0.648 (0.605-0.691)	0.628 (0.555-0.697)
	CR + Cluster-M	0.651 (0.609-0.692)	**0.637 (0.573-0.701)**
	CR + Dist.	0.563 (0.467-0.609)	0.534 (0.46-0.604)
	CR + Cluster-C + Cluster-M	0.667 (0.626-0.710)	0.627 (0.557-0.697)
	CR + Cluster-Max + Dist	0.658 (0.614-0.702)	0.623 (0.555-0.69)
	CR + Cluster-C + Dist	0.655 (0.610-0.699)	0.615 (0.539-0.689)
	CR + Cluster-C + Cluster-M + Dist	**0.672 (0.628-0.717)**	0.615 (0.546-0.681)

NOTE.- AUCs are given with 95% confidence intervals. Other abbreviations: CR: clinical-radiological input, Dist.: range of lesion-to-centroid Euclidean distances.
§: input variables can be found in the multivariable analyses of Table 3.

SUPPLEMENTAL TABLE ST7. Associations of radiomics clustering and distance-based metrics with patient outcome, i.e., objective response rate to the first line therapy and overall survival.

Characteristics	Objective response rate to 1st line					Overall survival					
	No	Yes	*P*-value	univariable OR (95%CI)	*P*-value	No.at risk	No. Of events	Survival probability at 12 months	Log rank P-value	Univariable HR (95%CI)	*P*-value
**All patients**											
**Cluster-M**			**.026***						**.047***		
Cluster-M3+M4 (ref.)	113/212 (53.3)	63/148 (42.6)		-	-	176	152	50 (43.1-58.0)		-	-
Cluster-M1	34/212 (16)	19/148 (12.8)		1 (0.53-1.9)	.994	53	35	47.2 (35.5-62.7)		0.98 (0.67-1.41)	.896
Cluster-M2+M5	65/212 (30.7)	66/148 (44.6)		1.82 (1.15-2.89)	**.011***	132	80	62.2 (54.4-71.2)		0.71 (0.54-0.94)	**.016***
**Cluster-C**			.312						.387		
Cluster-C2 (ref.)	84/212 (39.6)	49/148 (33.1)		-	-	133	113	50.1 (42.3-59.4)		-	-
Cluster-C1	96/212 (45.3)	79/148 (53.4)		1.41 (0.89-2.24)	.144	176	109	57.0 (50.1-64.8)		0.83 (0.64-1.09)	.175
Cluster-C3	32/212 (15.1)	20/148 (13.5)		1.07 (0.55-2.07)	.838	52	45	53.8 (41.9-69.3)		0.95 (0.67-1.34)	.779
**Range of euclidean lesion-to-centroid distances (continuous)**	3.28 ± 2.69	2.6 ± 2.01	**.040***	0.89 (0.81-0.97)	**.010***	-	-	-	-	1.1 (1.04-1.15)	**<.001*****
**Range of euclidean lesion-to-centroid distances (binary)**			.335						**.027***		
< Median	101/212 (47.6)	79/148 (53.4)		-	-	180	125	56.3 (49.5-64.1)		-	
≥ Median	111/212 (52.4)	69/148 (46.6)		0.79 (0.52-1.21)	.284	181	142	51.7 (44.9-59.5)		1.31 (1.03-1.67)	**.028***
**Patients treated with CPI (± chemotherapy)**											
**Cluster-M**			.624								
Cluster-M3+M4 (ref.)	12/82 (14.6)	15/73 (20.5)		-	-	42	32	64.3 (51.3-80.5)	.583	-	-
Cluster-M1	65/82 (79.3)	54/73 (74)		0.66 (0.29-1.54)	.341	43	27	51.2 (38.2-68.5)		1.08 (0.64-1.82)	.781
Cluster-M2+M5	5/82 (6.1)	4/73 (5.5)		0.64 (0.14-2.92)	.564	70	42	61.4 (51.0-74.0)		0.84 (0.53-1.35)	.480
**Cluster-C**			.505								
Cluster-C2 (ref.)	21/82 (25.6)	21/73 (28.8)		-	-	27	20	66.7 (51.1-87.0)	.859	-	-
Cluster-C1	26/82 (31.7)	17/73 (23.3)		0.65 (0.28-1.55)	.333	119	74	57.1 (48.9-66.8)		1.09 (0.66-1.82)	.729
Cluster-C3	35/82 (42.7)	35/73 (47.9)		1 (0.47-2.15)	>.999	9	7	66.7 (42.0-100)		1.27 (0.53-3)	.593
**Range of euclidean lesion-to-centroid distances (continuous) +SD**	2.91 ± 2.3	2.4 ± 1.81	.203	0.89 (0.76-1.04)	.136	-	-	-	-	1.05 (0.96-1.15)	.323
**Range of euclidean lesion-to-centroid distances (binary)**			.570								
< Median	39/82 (47.6)	39/73 (53.4)		-	-	78	49	61.5 (51.6-73.3)	.225	-	

≥ Median	43/82 (52.4)	34/73 (46.6)		0.79 (0.42-1.49)	.466	77	52	57.1 (47.1-69.3)		1.27 (0.86-1.89)	.228
**Patients treated with TKI**											
**Cluster-M**			.117						.981		
Cluster-M3+M4 (ref.)	5/14 (35.7)	8/32 (25)		-	-	18	14	77.8 (60.8-99.6)		-	-
Cluster-M1	5/14 (35.7)	21/32 (65.6)		2.62 (0.6-11.57)	.202	4	2	75 (42.6-100)		1.13 (0.24-5.18)	.879
Cluster-M2+M5	4/14 (28.6)	3/32 (9.4)		0.47 (0.07-3.04)	.427	25	9	90.7 (79.2-100)		0.96 (0.39-2.39)	.938
**Cluster-C**			.305						.786		
Cluster-C2 (ref.)	7/14 (50)	11/32 (34.4)		-	-	13	9	66.7 (44.7-99.5)		-	-
Cluster-C1	2/14 (14.3)	2/32 (6.2)		0.64 (0.07-5.61)	.684	27	10	87.8 (75.8-100)		0.75 (0.29-1.94)	.559
Cluster-C3	5/14 (35.7)	19/32 (59.4)		2.42 (0.62-9.49)	.206	7	6	100 (100-100)		0.74 (0.26-2.09)	.565
**Range of euclidean lesion-to-centroid distances (continuous) +SD**	2.97 ± 2.87	2.19 ± 1.38	0.574	0.82 (0.59-1.13)	.218	-	-	-	-	1.31 (1.09-1.59)	**.004****
**Range of euclidean lesion-to-centroid distances (binary)**			0.671						**.035***		
< Median	8/14 (57.1)	22/32 (68.8)		-	-	30	15	89.3 (78.5-100)		-	-
≥ Median	6/14 (42.9)	10/32 (31.2)		0.61 (0.17-2.21)	.449	17	10	75 (56.5-99.5)		2.34 (1.02-5.35)	**.044***

NOTE.- *: *P*<.05, **: *P*<.005, ***: *P*<.001. Significant results are in bold. Abbreviations: 95%CI: 95% confidence interval, CPI: checkpoint inhibitor, HR: hazard ratio, no.: number, ref.: level of reference, TKI: tyrosine kinase inhibitor, WHO-PS: World health organization performans status.

SUPPLEMENTARY TABLE ST8. Summary of the multivariable survival analyses to predict overall survival in the entire population and in patients treated with tyrosine kinase inhibitors (TKI).

Characteristics	Multivariable HR (95%CI)	*P*-value
**All patients**		
**Cluster-M**		
Cluster-M3+M4 (ref.)	-	-
Cluster-M1	1.05 (0.71-1.53)	0.8160
Cluster-M2+M5	0.80 (0.60-1.06)	0.1208
**Range of euclidean lesion-to-centroid distances (continuous)**	1.07 (1.01-1.12)	**0.0143***
**WHO PS**		
**PS = 0 (ref.)**	-	**-**
**PS = 1**	1.27 (0.94-1.73)	0.1232
**PS = 2**	3.41 (2.39-4.87)	**<0.0001*****
**Sex = women (ref: men)**	1.04 (0.80-1.34)	0.7763
**Age at diagnosis (continuous)**	1.01 (0.99-1.02)	0.0654
**Initial staging = IVB (ref: other)**	1.46 (1.05-2.02)	**0.0229***
**Patients treated with TKI**		
**Range of euclidean lesion-to-centroid distances (continuous)**	1.33 (1.09-1.63)	**0.0055***
**WHO-PS**		
**PS = 0 (ref.)**	**-**	**-**
**PS = 1**	0.90 (0.36-2.24)	0.82261
**PS = 2**	4.25 (0.94-19.31)	0.06083
**Sex = women (ref: men)**	1.38 (0.56-3.37)	0.48179
**Age at diagnosis (continuous)**	1.03 (0.99-1.06)	0.19422
**Initial staging = IVB (ref: other)**	2.60 (0.57-11.96)	0.21959

NOTE. – Only radiomics derived features associated with overall survival in the univariable analyses were included in this multivariable analysis.
*: *P*<.05, **: ***: *P*<.001. Significant results are in bold. Abbreviations: 95%CI: 95% confidence interval, HR: hazard ratio, ref.: level of reference, TKI: tyrosine kinase inhibitor, WHO-PS: World health organization performans status.
